# A deep learning method for empirical spectral prediction and inverse design of all-optical nonlinear plasmonic ring resonator switches

**DOI:** 10.1038/s41598-024-56522-3

**Published:** 2024-03-09

**Authors:** Ehsan Adibnia, Mohammad Ali Mansouri-Birjandi, Majid Ghadrdan, Pouria Jafari

**Affiliations:** Faculty of Electrical and Computer Engineering, University of Sistan and Baluchestan (USB), P.O. Box 9816745563, Zahedan, Iran

**Keywords:** Deep learning, Artificial neural network, Plasmonic switch, Inverse design, Nanophotonics, Applied optics, Computer science

## Abstract

All-optical plasmonic switches (AOPSs) utilizing surface plasmon polaritons are well-suited for integration into photonic integrated circuits (PICs) and play a crucial role in advancing all-optical signal processing. The current AOPS design methods still rely on trial-and-error or empirical approaches. In contrast, recent deep learning (DL) advances have proven highly effective as computational tools, offering an alternative means to accelerate nanophotonics simulations. This paper proposes an innovative approach utilizing DL for spectrum prediction and inverse design of AOPS. The switches employ circular nonlinear plasmonic ring resonators (NPRRs) composed of interconnected metal–insulator–metal waveguides with a ring resonator. The NPRR switching performance is shown using the nonlinear Kerr effect. The forward model presented in this study demonstrates superior computational efficiency when compared to the finite-difference time-domain method. The model analyzes various structural parameters to predict transmission spectra with a distinctive dip. Inverse modeling enables the prediction of design parameters for desired transmission spectra. This model provides a rapid estimation of design parameters, offering a clear advantage over time-intensive conventional optimization approaches. The loss of prediction for both the forward and inverse models, when compared to simulations, is exceedingly low and on the order of 10^−4^. The results confirm the suitability of employing DL for forward and inverse design of AOPSs in PICs.

## Introduction

In recent decades, significant progress in the realm of nanophotonics has surpassed the limitations imposed by diffraction, leading to remarkable progress. These advancements have found diverse applications in domains such as biology and nanotechnology. Specifically, the emergence of nanophotonics has revolutionized optics by enabling precise control over the interaction between light and matter through the utilization of subwavelength structures^1–3^. AOPSs are an essential nanophotonic technology widely utilized in integrated optical circuits and all-optical networks^4^. Among proposed optical switch architectures operating under distinct principles and exhibiting unique characteristics, structures leveraging SPP excitation have attracted significant research attention due to their rapid response times, low power demands, and nanoscale dimensions^5,6^. SPPs are electromagnetic waves propagating at metal–dielectric interfaces, with the caveat that solely transverse magnetic (TM) polarized light can stimulate SPPs in metallic media. Illuminating metal nanostructures with TM-polarized radiation induces SPP excitation^7^. Owing to their nanometric footprint, plasmonic switches offer fast switching speeds, low operating powers, and high transmission efficiency. A particularly interesting approach for realizing plasmonic switches involves harnessing the optical nonlinear Kerr effect^8,9^. optical cavities^10,11^ and ring resonators^12^ are common techniques for implementing such Kerr effect-based plasmonic switches.

In nanophotonics, the objective is to utilize optical resonances and intense localized fields generated by surface plasmons. This can be achieved by optimizing nanostructure shapes or selecting suitable materials. However, dealing with complex nanostructures that involve multiple geometric factors necessitates advanced numerical techniques.

Forward calculations in nanophotonics involve predicting the optical response through the solution of Maxwell’s equations, employing numerical methods such as the finite element method (FEM)^13^ and the finite-difference time-domain (FDTD) method^14^. Nonetheless, these methods often entail significant computational costs, demanding substantial memory resources and time-consuming simulations. Inverse design, which aims to create nanostructures with specific desired optical responses, poses a considerable challenge, even with advanced numerical tools. This challenge stems from the highly nonlinear nature of the problem and the complex interrelation among the independent properties of nanostructures and their associated optical responses. Solving inverse design problems in photonic devices typically requires extensive research efforts^15^. For readers seeking a broader understanding of inverse design approaches in photonics, we recommend referring to recent review articles that offer comprehensive overviews of this rapidly evolving field^16–19^.

To overcome the limitations machine learning (ML) techniques have arisen as a crucial tool. However, despite the simplicity and speed of ML-based regression methods, they often encounter challenges in meeting the generalization requirements of complex tasks^20^. The associated challenges related to achieving satisfactory generalization tasks remain prominent in the field of ML. The swift advancement of artificial intelligence (AI) has propelled DL as a state-of-the-art approach in the domain of regression. In contrast to traditional ML techniques, DL leverages the distinctive multi-layer architecture of neural networks (NNs) to extract information on features at various scales and deep levels, leading to improved regression accuracy and efficacy^21^. As a result, a growing number of scholars are utilizing DL methods in regression research^22^. DL methods show significant potential across diverse applications^21,23^, yet they face inherent limitations and challenge^24^.

These methods commonly rely on large amounts of data for training, which requires considerable time and resources for data acquisition and preparation^25^. The quality and representativeness of the data are crucial for accurate predictions, as inadequate or imbalanced data can lead to suboptimal results^26^. Generalization poses another challenge, as DL models excel at recognizing patterns in training data but struggle to extend this capability to unseen or significantly different scenarios^26^. Overfitting, where models become overly specialized to training data, often reduces performance on new instances^27^. Additionally, the interpretability of DL models remains a formidable obstacle due to their opaque nature, hindering understanding of the decision-making process and impacting trust, particularly in critical applications^28,29^. Ongoing research aims to unravel factors contributing to predictions and provide transparent explanations^30^. Furthermore, the use of DL models, especially complex architectures, demands substantial computational resources for training and inference, necessitating specialized hardware like GPUs or TPUs^31,32^. Deploying large-scale models in resource-constrained environments poses additional challenges^33^. Lastly, DL models are susceptible to adversarial attacks, where carefully crafted perturbations to input data can lead to misclassification or erroneous predictions^32–34^. Ensuring the robustness and security of DL models against such attacks is an active research area. Addressing these limitations and challenges requires persistent research and development efforts in the field of DL, along with meticulous consideration of contextual factors and potential drawbacks when applying these methodologies in practical applications. The following paragraph discusses several significant studies that have employed DL techniques in the realm of photonics.

### Related works

The past 10 years have seen the emergence of DL, which has had an unparalleled impact on numerous research areas. The field of photonics has similarly reaped the rewards of the swift progress in DL methods^35^ such as multilayer perceptrons (MLPs)^36^, convolutional neural networks (CNNs)^37^, variational autoencoders (VAEs)^38^, recurrent neural networks (RNNs)^39^, and generative adversarial networks (GANs)^40^. A CNN is perfect for data processing in photonics research as it easily handles data in high-dimensional spaces, such as the photonic patterns rendered as images, and the spectral responses from specific photonic devices^35^. Take for example the work of Asano and his team^41^ who leveraged CNN to fine-tune the locations of nanocavities, thereby substantially amplifying the Q-factor from 3.8 × 10^8^ to 1.6 × 10^9^. The application of Variational Autoencoders (VAEs) aids in the simplification of the design space and the optical response features in photonic devices, thereby furnishing crucial knowledge regarding light-matter interaction that is indispensable for the enhancement of the device^35^. Leveraging the reduced dimension and continuity of the latent space, Ma et al.^42^ used of a VAE-based generative model, the meta-atoms of a double-layered chiral metamaterial and its corresponding optical responses were successfully encoded. This enabled a deep investigation into the complex relationship between their structure and performance, eliminating the need for extensive data collection. RNNs represent a distinct category of DL models skillfully addressing issues related to sequence data like phrases and audio signals^39^. RNNs are remarkably fitting in imagining optical signals or spectra in the time domain with a distinctive line shape caused by various resonance modes while designing photonics^35^. Zhou and co-workers^43^, have effectively employed RNNs to scrutinize optical signals and noise mitigation in high-speed fiber transmission. The GAN represents a different type of deep generative model, assembled with the combination of a generator and discriminator^39^. The generator undergoes training in a counter-operative way with the aim to produce samples that ideally construct a distribution that cannot be distinguished from the training dataset^39^. GANs, capable of creating large nanostructures quickly, have been employed to stochastically design and enhance dielectric and metallic metasurfaces^35^. For example, a GAN-based framework for the reverse engineering of metasurface nanostructures to meet specific design requirements has been proposed by Liu and his associates^44^. In a recent publication, Chen and colleagues^45^ have unveiled an innovative approach that employs a transformer-based model, designated as photonic vision transformer (POViT), for the efficient design and simulation of photonic crystal nanocavities. This method addresses the exigent task of achieving swift and accurate characterization of nanoscale photonic devices with minimal human intervention. Notwithstanding its relatively straightforward architecture, the MLP model's capacity to adequately fit any continuous functions using a finite number of neurons has been theoretically confirmed^46^. Prior to the remarkable triumphs of DL within the field of computer vision, scholars had begun to investigate the application of MLPs within photonics^35^. The efficacy of these models was considerably constrained due to nascent training strategies, data scarcity, and consequently, the limited complexity of the model architectures^36^. In the past few years, with the amelioration of earlier limitations and guided by the contemporary consensus within the artificial intelligence community, which favors the utilization of less complex NN frameworks when they are adequate for the successful execution of the given task, the MLP has reclaimed its prominence among researchers in the field of photonics^35^. For instance, Tahersima et al.^47^ suggested employing DL to construct SOI-based 1 × 2 powers splitters that have different targeted transmission ratios at a pair of ports. Here, the array of binary variables embodies the device structure, revealing whether the silicon is etched at specific coordinates or not.

The NNs have been successfully employed to approximate various physics simulations, as demonstrated by Carleo et al.^48^, who utilized NNs to solve complex many-body quantum physics problems. Several independent scholarships have shown the effectiveness of NN-based methods in learning the correlation between device geometry and optical behavior. For instance, Peurifoy et al.^49^ employed NNs to estimate the phenomenon of light scattering by nanoparticles with multiple layers. One strength of their research lies in implementing inverse design for both specific and broadband wavelengths. Similarly, Suchowski et al.^50^ employed deep neural networks (DNNs) to predict nanostructure geometries solely based on far-field response. They also highlighted the utility of inverse design for chemical sensing applications. Brian Anthony et al.^51^ explored ML methods to predict the design parameters of metasurfaces, considering the desired electromagnetic field outcomes. They also developed a DL framework for mapping the design space of topological states in photonic crystals^52^. Baxter et al.^53^ demonstrated the application of DL in predicting plasmonic metal colors and in the inverse design of laser parameters, enabling an expansion of the available color options without the necessity for additional simulations or experiments. Through rigorous numerical simulations and extensive data training, Sajed et al.^54^ engineered a DNN model capable of accurately predicting absorption responses from images of plasmonic nanostructures. Zhang et al.^55^ proposed an approach based on artificial neural networks (ANNs) for spectrum prediction in plasmonic waveguide-coupled cavity structures. They employed genetic algorithms to facilitate the design of the network architecture and the selection of appropriate ANN hyperparameters. This approach combined the optimization capabilities of genetic algorithms, inspired by biological evolution, with the adaptability and learning capabilities of ANNs. In a recent publication, Verma et al.^56^ presented a DNN framework for predicting the transmission, reflection, and absorption spectra of two elliptical nanostructures, on a SiO_2_ substrate. The linear relationship (A = 1 − T + R) between the absorption spectrum (A), transmission spectrum (T), and reflection spectrum (R) enables the derivation of the absorption spectrum from the transmission and reflection spectra. Hence, it is worth mentioning that there was an opportunity to avoid using all three spectra as outputs for the NN. By leveraging this relationship, it would have been possible to utilize only the transmission and reflection spectra as inputs to the NN, thereby simplifying the model architecture and potentially reducing computational complexity.

DL in photonics faces challenges including the dependence on large datasets or pre-trained MLPs, the generation of non-unique designs from many-to-one mappings, and constraints on optimizing photonic structures beyond reverse engineering. In over 5 decades, reinforcement learning (RL) has advanced from a purely mathematical model to a highly effective solution for a growing number of actual real-world tasks with substantial scientific and engineering implications^57^. The remarkable achievement of RL in matching or outperforming human skills is clearly demonstrated by Google DeepMind’s AlphaGo Zero^58^ and AlphaStar^59^. RL stands out in the inverse problem as it doesn’t necessitate pre-collected training data, mitigates the non-uniqueness problem, and is capable of optimizing the photonic structure beyond the scope of inverse designing. Nonetheless, there is still a significant potential for exploration in the realm of RL. Pursuing this line of advancment, Sajedian and colleagues have recently, in two different studies, made use of deep reinforcement learning to enhance the optimization of dielectric nanostructures^60^ and high-transmission color filters^61^. This signifies the introduction of reinforcement learning in the realm of photonics design. Sui and associates^62^ have recently showcased the strong convergence of their model by utilizing deep reinforcement learning to invert the design of digital nano-materials. Through posing chip floor planning as a deep RL problem, Mirhoseini et al.^63^ have enhanced the design of Google's latest tensor processing unit accelerators, exceeding the efficiency of the most powerful baseline models. The application of a deep RL approach in the management of dissipative solitons generation in a mode-locked fiber laser system was successfully illustrated by Kuprikov and his team^64^. In a study, Li et al.^65^ have developed learning to design optical-resonators (L2DO), an approach using reinforcement learning to autonomously inverse design unique nanophotonic laser cavities without prior knowledge, significantly outperforming traditional methods and human expertise in both design quality and computational efficiency.

### Research gap

It is well-established among photonics researchers that the ring resonator (RR) is one of the fundamental building blocks of integrated photonic circuits. As a basic resonant element, RRs underpin numerous photonic applications requiring spectral filtering, modulation, and sensing^8,66,67^. Moreover, intensity-dependent switching functionality—enabled through nonlinear optical effects in resonators—provides one of the primal operations upon which many advanced photonic devices are constructed. While significant advances have been achieved applying DL techniques for regression problems in nanophotonics, an identified gap remains in leveraging DL for predicting spectra and inverse designing of AOPS utilizing RRs. Given the critical importance of RRs within PICs, it is imperative to capitalize on the benefits of DL to address remarkable challenges in spectrum prediction and inverse design methodologies for these devices. While nascent applications of ML have begun to emerge, a comprehensive framework focusing specifically on all-optical switching regimes has been lacking. The research presented herein thus aims to systematically investigate filling this void through innovative DL-driven approaches customized for plasmonic resonator-based switching structures. The subsequent paragraph delineates the objectives and contributions of this research.

### Objectives and contributions

In this manuscript, we focus on the significant research interest surrounding AOS based on Surface SPPs due to their ultra-fast response time and nanoscale characteristics. In undertaking this work, we have leveraged our prior investigation efforts and experiences related to the design, simulation, and analysis of AOPSs. Specifically, we build upon the proposed nanophotonic structure introduced in our previous study^12^ to serve as a testbed for exploring the application of DL techniques to this domain.

In this paper, the intersection of nanophotonics and artificial intelligence is a key focal point. Motivated by the impactful previous studies discussed in the related works segment, the present research aspires, to the best of our knowledge, to pioneer the demonstration of the potential of DL in predicting spectra and conducting the inverse design of AOS based on NPRR. Our primary objective is to propose a cutting-edge methodology that incorporates DL for spectrum prediction. This approach aims to enhance our understanding of the characteristics of all-optical NPRR switches and facilitate their inverse design.

By leveraging DL techniques, we aim to develop a predictive model capable of accurately predicting the spectral response of these devices. This innovative methodology shows potential in advancing the domain of AOPS by aiding in design optimization and providing deeper insights into the fundamental principles, behind their operation. We demonstrate the effectiveness of our proposed NPRR through the use of the Kerr effect and evaluate the performance of our suggested switches using the FDTD technique. In this approach, an ANN is initially trained to approximate simulations and accurately predict transmission spectra giving us an understanding of the resonating wavelength characteristics of devices. While our main focus is addressing AOS problems the approach, we present in this study can easily be extended to effectively tackle other nanophotonic challenges.

To establish a coherent structure for this manuscript, we offer a concise outline of the subsequent sections. The “Theory” section will encompass the fundamental principles underlying AOPS design, as well as the formulation of the NN problem, with a specific emphasis on the utilization of DL techniques for spectrum prediction and inverse design. The “Method” section will delve into the methodology employed in this investigation, encompassing FDTD simulation, data generation, and preprocessing, as well as the procedure for training the DL models. In the subsequent section, we will present and discuss the outcomes of the forward and inverse models, followed by a comprehensive analysis. Lastly, in the “Conclusions” section, we will summarize the key findings and conclude.

## Theory

In light of the interdisciplinary nature of the study, the theoretical framework of this paper is bifurcated into two subsections within the “Theory” section. The initial sub-section delves into the equations dictating the design and modeling of AOPS, whereas the subsequent subsection centers on the development of the NN formulation.

### AOPS design and modeling

**Figure 1:**
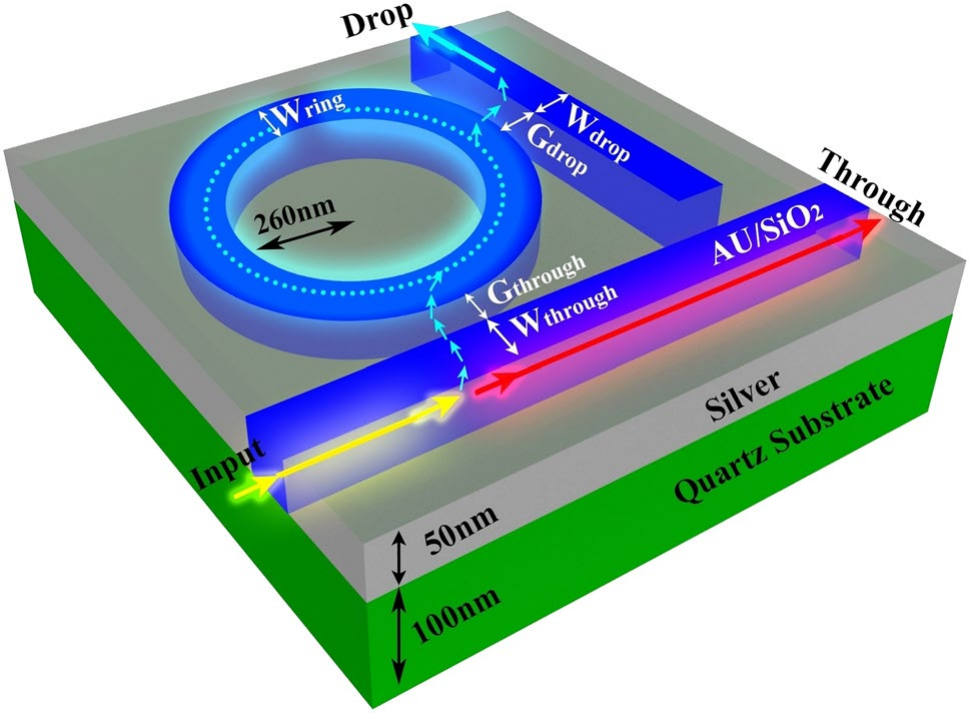
The schematic of the circular AOPS configuration. This three-dimensional schematic provides a visual representation of the geometrical structures and parameters employed in AOPS design.

As alluded to earlier in this article, we have drawn upon previous achievements in the design and implementation of AOPSs. In our AOPS design, we have incorporated a configuration and materials that have consistently exhibited outstanding performance in the context of switching applications. Furthermore, current fabrication technology is fully capable of realizing this design^12,68^. This configuration involves a circular RR connected to input and output waveguides with varying widths. The AOPS structures, depicted in Fig. 1, consist of a silver film with a layered structure featuring a low refractive index material comprised of Au/SiO_2_. Within this configuration, we utilize an Au/SiO_2_ composite as the nonlinear material, which possesses a substantial Kerr nonlinearity. The composite exhibits a refractive index of *n*_0_ = 1.47, and its Kerr nonlinear coefficient is *n*_2_ = 2.07 × 10^−9^ cm^2^/W^69^.

The composite of Au/SiO_2_ is formed by incorporating gold particles into glass. These metal particles give rise to surface plasmon resonance, which can potentially result in an increased third-order optical nonlinear susceptibility^70^. The cladding layer encompassing the AOPS is composed of air, while the substrate is fabricated from glass. To ensure practicality in terms of fabrication technology, both the silver and Au/SiO_2_ layers exhibit a thickness of 50 nm. Additionally, a glass film with a thickness of 100 nm is employed as the substrate. We can calculate the resonant wavelengths, λ_*r*_, using the following equation^71^:

1 $$ {\uplambda }_{{\text{r}}} = Ln_{{{\text{eff}}}} /m $$

where *L* represents the effective length, *n*_eff_ is the effective refractive index, and *m* denotes an integer value. The nonlinearity observed in the RRs is attributed to the Kerr effect. The refractive index is dependent on the Kerr nonlinear coefficient and the intensity of the incident light, as described in reference^72^.

2 $$ n = n_{0} + n_{2} I $$

where *n*_0_ represents the linear refractive index, *n*_2_ is the nonlinear refractive index coefficient, and *I* denotes the light intensity.

SPPs in metallic structures are predominantly excited through transverse magnetic (TM) polarization^73^. SPPs exhibit distinct features, such as substantial electromagnetic field amplification, localization at subwavelength scales, and heightened sensitivity to the adjacent dielectric environment. These inherent attributes make SPPs particularly appealing for various applications, such as nanophotonics^74,75^, metamaterials^76^, and biosensing^77,78^. TM-polarized light interacting with a metallic structure generates SPPs at the metal interfaces. Moreover, when TM-polarized light is incident at the resonant wavelength, it triggers the excitation of SPPs within the RR structure. In this study, we consider silver as the metal in this configuration, and its complex dielectric constant is described using the Drude model^68,79^.

3 $$ \varepsilon \left( \omega \right) = \varepsilon_{\infty } - \omega_{{\text{p}}}^{2} /\left( {\omega^{2} - j\gamma_{{\text{p}}} \omega } \right) $$

where *ε*_∞_ = 1.95 represents the relative permittivity at infinite frequency, *ω* is the angular frequency of the incident lightwave, γ_p_ = 2 × 10^13^ rad/s is the electron collision frequency, and *ω*_p_ = 1.37 × 10^16^ rad/s is the plasma frequency. It is noteworthy that the dielectric constant can be broken down into its real and imaginary components (refer to Supplementary Eqs. S1 and S2 online for more details). The Drude–Lorentz model finds applications in various areas, including the characterization of electronic transitions in metals and phenomena related to surface plasmon resonance. For a more detailed analysis and explanation of the Drude–Lorentz model's application in these contexts, please refer to Sect. S1 of the Supplementary Information.

### DNN formulation

After comprehending the geometric parameters of the AOPS depicted in Fig. 1, we now proceed to present the mathematical formulation of the forward and inverse models. These models are essential in defining the problem for the NN by establishing a relationship between the input variables and the resulting transmission spectra.

Given the geometric design parameters G_*i*_ for the AOPS, we establish the forward model to calculate the transmission spectrum for both the through port and drop port. The transmission spectra T_1_ and T_2_ in the through port and drop port, respectively, can be obtained using the mapping function **F** and the input variables:

4 $$ {\text{T}}_{j}^{\lambda } = {\mathbf{F}}\left( {{\text{G}}_{1} = {\text{W}}_{{{\text{through}}}} ,{\text{ G}}_{2} = {\text{W}}_{{{\text{drop}}}} ,{\text{G}}_{3} = {\text{W}}_{{{\text{ring}}}} ,{\text{G}}_{4} = {\text{G}}_{{{\text{through}}}} ,{\text{G}}_{5} = {\text{G}}_{{{\text{drop}}}} ,{\text{G}}_{6} = {\uplambda } } \right) $$

where W_through_ is through waveguide width, W_drop_ is drop waveguide width, W_ring_ is ring waveguide width, G_through_ is through waveguide gap, G_drop_ is drop waveguide gap and λ is wavelength of input light, ranging λ = 1000–1800 nm.

In this mathematical framework, through the incorporation of the input light's wavelength as an input variable into the NN’s forward model, we have endowed it with the capacity to predict the transmittance values at both discrete single wavelengths and broadband of wavelengths. To find the optimal estimator for the forward model, we define an objective function as follows:

5 $$ \mathop {\min }\limits_{{i = {\text{G}}_{1} ,{\text{G}}_{2} , \ldots ,{\text{G}}_{6} }} \left\{ \left\| { {{\hat{\mathbf{F}}}\left( {{\text{G}}_{i} } \right) - {\text{g}}_{{\text{T}}} }} \right\| \right\} $$

In the equation above, ${\hat{\mathbf{F}}}$ represents the predicted function, while the symbol ${\text{g}}_{{\text{T}}}$ denotes the ground truth or actual observed values (transmission spectra). Thus, the DL problem involves finding the optimal estimator for ${\hat{\mathbf{F}}}$ based on the available training data.

Alternatively, an inverse problem aims to reconstruct the design space, from the given transmission values. Instead of relying on conventional methods, we introduce a unique strategy. We define a design space, from the transmission spectra as seen in Eq. (6), Where, T_*j*_ is transmission spectra. This can be achieved through the following inverse function:

6 $$ {\text{G}}_{i} = {\mathbf{I}}\left( {{\text{T}}_{j} } \right) $$

To identify the most suitable estimator for the inverse model, we formulate an objective function in the following manner:

7 $$ \mathop {\min }\limits_{{i = {\text{T}}_{1} ,{\text{T}}_{2} }} \left\{    \left\| {{\hat{\mathbf{I}}}\left( {{\text{T}}_{j} } \right) - {\text{g}}_{{\text{G}}} } \right\|\right\} $$

where ${\hat{\mathbf{I}}}$ symbolizes the predicted function, whereas ${\text{g}}_{{\text{G}}}$ denotes the ground truth values (Geometric parameters).

What sets our research apart is the recognition of a common challenge in the field. Traditional analytical methods often fall short when attempting to derive the inverse function for AOPS configurations. In these cases, we introduce a novel perspective by advocating for the use of DL techniques as an alternative solution. DL offers a powerful means of non-linear approximation, and by adopting this approach, our research pioneers a more effective path to address both the forward and inverse models. This innovative methodology leads to a comprehensive understanding of the intricate behavior of AOPS, marking a distinctive contribution to the field. Following a review of the theoretical underpinnings, the subsequent section will undertake an examination of the methodology employed in this work.

## Method

The “Method” section encompasses two distinct subsections. The primary subsection will elucidate the process of data generation and preparation. Subsequently, the second subsection will delineate the procedure for training the NN.

### Generation and preprocessing of data

To investigate the performance of the AOPS, we select design parameters carefully. These parameters include the through waveguide width (W_through_), drop waveguide width (W_drop_), ring waveguide width (W_ring_), through waveguide gap (G_through_), and drop waveguide gap (G_drop_). Within our study, we systematically vary the values of W_*t*hrough_, W_drop_, and W_ring_ between 30 and 60 nm, while adjusting G_through_ and G_drop_ between 15 and 25 nm.

The training dataset was generated using FDTD simulations and involved extensive experimentation with the combination of different input parameters. For more detailed information regarding the composition of the dataset, including the distribution of input and output variables, please refer to Sect. S2 of the Supplementary Information. Figure S2 illustrates a relatively balanced assortment of data points across the entire domain. The well-distributed uniformity of the data in Fig. S2 provides strong evidence for the robustness and reliability of the dataset. It ensures that the dataset covers all input and output dimensions in a representative and comprehensive manner, thus enabling accurate and meaningful analysis.

In the past, constructing the dataset using the FDTD method was a time-consuming process. It would take several minutes to calculate the transmission spectrum for a single structure. As a result, when dealing with a significantly larger dataset, the processing time would become impractically long. A comprehensive analysis of the computational cost has been provided in Sect. S3 of the Supplementary Information. Section S3 thoroughly discusses the analysis of computational cost, including a detailed comparison of execution times for different meshing strategies (refer to Supplementary Fig. S4 online). It is important to note that generating large datasets for training DL models, especially when using computationally intensive methods like electromagnetic simulations, can initially incur significant computational costs. However, it is crucial to view this data generation process as a one-time investment that establishes the foundation for more efficient predictions and analyses in the future.

The entire process of generating the dataset carried out on three parallel computers, each with a 2.9 GHz Intel processor boasting 16 cores, took about 34 days to complete. This time-consuming data generation stage reflects the significant computational demands associated with producing a large dataset for training. The input to the NN comprised standardized design parameters of the AOPS, while the output consisted of the normalized spectrum sampled at 800 points within the wavelength range of 1000–1800 nm. However, the trained NN model successfully overcomes this challenge by predicting hundreds of thousands of datasets in less than 1 min, resulting in a significant reduction in processing time compared to the lengthy data generation process. This highlights the efficiency and effectiveness of the trained model in accelerating the analysis and prediction of AOPS performance. Considering the continuous nature of the wavelength quantity, the selection of 800 points for sampling the transmission spectrum, which equates to choosing a 1-nm sampling increment, will not pose an issue regarding the inclusion of all possible physical states within the training dataset. This sampling strategy is selected to circumvent the computational complexities associated with fractional wavelengths and to optimize memory efficiency for our datasets.

In order to enable the uniform and automated generation of the expansive training dataset requisite for this study, a fixed grid with a resolution of 2 nm was utilized for the data generation process. This resolution was selected after careful consideration of multiple factors pertinent to the aims and scope of our research. Firstly, a spacing of 2 nm provides an optimal balance between retaining the level of spatial detail imperative to capture the nanoscale physical phenomena central to this work, while also maintaining computational tractability across the parallel processing resources utilized for dataset creation. Secondly, comprehensive preliminary analysis indicated that a 2-nm resolution suffices to reveal the patterns and behaviors of interest to the desired degree of accuracy, without necessitating finer grids that would substantially increase computational overheads without proportional gains in analytical capability.

Through meticulous selection and generation of this extensive dataset, we can proficiently train our NN model to precisely forecast the transmission spectra of the AOPS across a diverse array of design parameter combinations. This capability empowers us to acquire invaluable insights into the optimal functioning of the AOPS across various operational scenarios.

### Procedure of training

The NN implementation in this study utilizes Keras, a high-level NN library integrated into Google's TensorFlow framework in 2016. The calculations are performed in the Spyder Python environment (Python version 3.7.13 and Spyder version 5.1.5) within Anaconda (version 4.14.0). To develop and train the DNN, we conducted an extensive evaluation of various ML packages.

The data preprocessing stage involved using pandas^80^, while Scikit-learn^81^ was employed for intensive training. NumPy^82^, known for its advanced capabilities in handling matrices and multidimensional arrays, played a significant role in constructing the regression model.

To enhance the performance of the NN during the validation stage, a systematic exploration of hyperparameters was undertaken. This involved fine-tuning the learning rates and adjusting the neuron count per layer to identify the optimal configuration. The dataset was partitioned into three distinct categories: training, validation, and testing, with respective proportions of 70%, 15%, and 15%. It was then trained using a batch size of 80. Given that a single FDTD simulation can assess all frequency points simultaneously, it is critical to adopt a data-splitting approach that mirrors real-world conditions. In an inverse modeling context, a designer would only simulate designs that have not been previously computed. Therefore, the data partitioning should occur at the level of the design variables, exclusive of the wavelength, to maintain the integrity of real-world application scenarios. Throughout the training process, the model's parameters were updated by computing gradients based on the training loss. The training process was halted when the validation loss no longer displayed any further improvement. Multiple architectures and models were evaluated, and the model exhibiting the minimum validation loss was selected. It is vital to emphasize that all the figures presented in this paper were derived from the validation set, which was not utilized for model training. To ensure a rigorous assessment of the model's performance, we have exclusively allocated a comprehensive subset of physical design configurations, along with all their associated wavelength points, to the test set. This will allow us to evaluate the model's performance on entirely new designs, providing a more stringent test of its generalization capabilities. However, the model was optimized to deliver satisfactory performance on the validation set, indicating its competence in generalizing to unseen data.

The AdaDelta Optimizer was utilized in our approach to search for local or global maxima/minima within the design space^83^. Choosing the optimal hyperparameters, including batch size, iteration numbers, and learning rate, was crucial in the training process. A learning rate of 0.1 was set, and 5000 epochs were performed. After 5000 epochs, the changes in both the training and validation loss became negligible, indicating that further iterations did not significantly improve the model's performance. We acknowledge that the aforementioned parameters were not extensively optimized, leaving room for the discovery of more efficient schemes. Exploring different hyperparameter configurations and optimization strategies could lead to improved results. Details on our efforts to optimize critical hyperparameters, including the number of hidden layers, neurons per layer, and trained epochs, can be found in Sect. S4 of Supplementary Information (refer to Supplementary Figs. S5 and S6 online). As previously mentioned, Sect. S3 of Supplementary Information provides additional information on the comprehensive analysis of computational costs. In Fig. S3, the computational costs associated with varying these hyperparameters in DL are illustrated. This figure demonstrates how changing these hyperparameters impacts the computational requirements of the learning process.

In terms of choosing the activation function, the current study diverges from the related works reviewed in the “Introduction” section, which predominantly used the ReLU function. To address gradient vanishing issues that can hinder the training of the network, this study employs the Leaky ReLU^84^ activation function. The ReLU function suffers from the problem of gradient vanishing when *x* < 0, which hinders parameter updates and affects classification accuracy. The Leaky ReLU function overcomes this problem by encompassing the entire range of real numbers and introducing a small linear component in the negative region.

This property allows Leaky ReLU to facilitate improved convergence during optimization. Furthermore, the nature of the continuous value estimation task performed herein, requiring continuous predictions, is well-suited to the Leaky ReLU function. Equation (8) provides the formula for the function^85^.

8 $$ {\text{Leaky ReLU}} = \left\{ {\begin{array}{*{20}l} {\alpha x} \hfill & {\quad x \le 0} \hfill \\ x \hfill & {\quad x > 0} \hfill \\ \end{array} } \right. $$

In this equation, $x$ represents the input value, and $\alpha$ is a small positive constant that determines the slope of the function for negative inputs.

We used the mean squared logarithmic error (MSLE) function for error estimation, which offers advantages over mean squared error (MSE) by providing improved accuracy for positive and continuous predicted values^86^. Mathematically, it can be defined as^87^:

9 $$ {\text{loss}} = \frac{1}{n}\mathop \sum \limits_{i = 1}^{n} \left( {\log \left( {1 + y_{{{\text{pred}}}} } \right) - \log \left( {1 + y_{{{\text{true}}}} } \right)} \right)^{2} $$

where *n* represents the number of data points, *y*_pred_ is the predicted value, and *y*_true_ is the true value calculated using the FDTD method.

We treated the choice of error function similar to other hyperparameters, evaluating options to determine the one yielding the lowest loss. Although differences between certain functions, such as MSE and MSLE, were slight, we aimed to select the function that minimized the loss value. Our rationale for opting to utilize the MSLE over alternative error metrics is elaborated in Sect. S4 of the Supplementary information.

The subsequent section will unveil the outcomes derived from the application of these methodologies, elucidating the degree of success in attaining the objectives outlined in this research.

## Results and discussion

The “Results and discussion” section of this article will be articulated through three discernible subsections. The initial subsection will scrutinize the outcomes derived from the training of the forward model and subject them to analysis. The second subsection will represent the model's efficacy in aiding designers of AOPS. Finally, the last subsection deliberates on outcomes related to the inverse model, spotlighting the remarkable proficiency of the DL-based approach in confronting a pivotal challenge within the realm of all-optical switch design, namely the challenge of inverse design.

### Forward DNN

**Figure 2:**
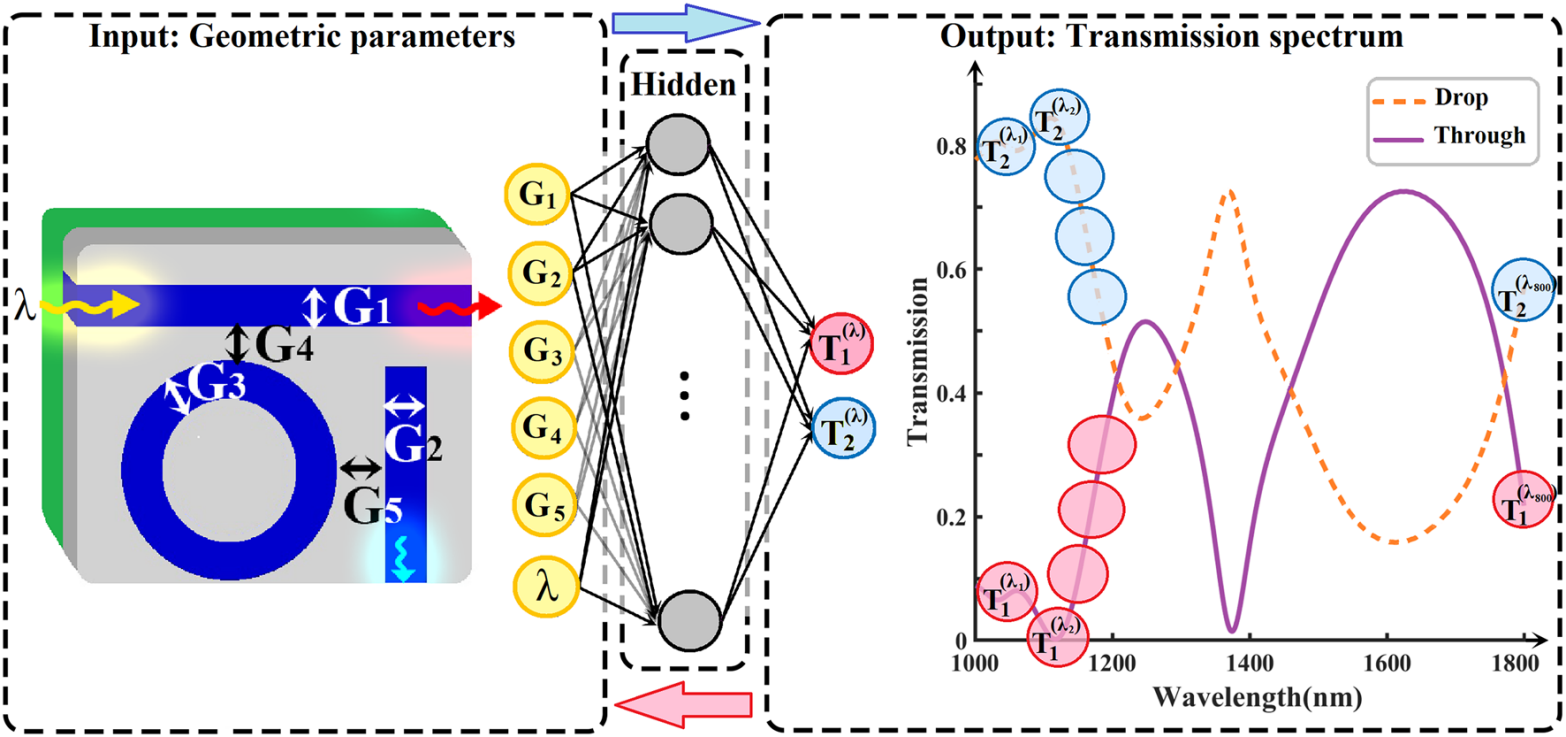
The NN architecture used for spectrum prediction in the AOPS takes as inputs the geometric parameters of the AOPS (waveguide width, gap between waveguides) and the wavelength of the input light. After passing through six hidden layers, the NN outputs the expected transmission across different wavelengths, representing the overall spectrum that would be obtained from the AOPS device. This NN architecture enables the capturing of complex relationships between the input parameters and the resulting transmission spectrum, providing valuable insights into the AOPS's behavior and performance.

In this portion, we evaluate the effectiveness of our approach in analyzing the transmission spectrum within an AOPS. Figure 2 presents the NN, which receives the geometric parameters of the RR as input. Delving into the exploration, we consider a spectrum of waveguide widths, spanning from 30 to 60 nm, and gap widths varying between 15 and 25 nm. This endeavor results in a rich training dataset, constituting 147,456 unique examples meticulously generated through FDTD simulations. Having a substantial amount of training data allows the DNN to be trained effectively. It can precisely model and predict millions of spectral characteristics of the RR structures within the specified parameter range.

Subsequently, we proceeded with training the NN using the generated dataset. The architecture of the NN, illustrated in Fig. 2, demonstrates how it utilizes input parameters to predict the spectral response of the corresponding AOPS. Our chosen architecture is a fully connected layer-based NN with optimized hidden layers of 6 and 60 neurons in each layer, resulting in a total of 114,665 parameters. In addition, within our model, the slope of the Leaky ReLU function, denoted as “$\alpha$”, is considered to be 0.2 (Eq. 8). The reasons for selecting this specific configuration are further explained in the Sect. S4 of Supplementary Information. Figure 2 showcases the output of the NN, which is the transmission spectrum sampled at various points ranging from 1000 to 1800 nm.

The methodological details, including the structure of the developed NN, the composition of the training dataset, selected learning rate, number of training iterations, activation functions employed, and loss metric computation are thoroughly explained in the “Method” section. To avoid redundancy and maintain brevity in the current context, we refrain from reiterating these details here. We encourage readers to refer to the Supplementary Information section for a detailed understanding of the specific methodologies employed in the study. Access to all source codes can be obtained from the following GitHub repository: https://github.com/ehsan20e20e/CircularRR_AOPS. This repository provides a comprehensive collection of code resources related to the generation of simulations, implementation of the model, and acquisition of results for the overarching issue addressed in this scholarly paper. The repository encompasses various code files, including MATLAB and Python scripts, which facilitate simulation execution, NN model training, and result analysis. Furthermore, detailed instructions are provided for executing the codebase within your computing environment.

**Figure 3:**
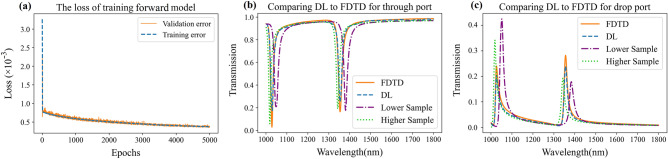
The results of the NN in approximating the spectrum. (**a**) The training loss is presented. The loss exhibits sharp declines, indicating that the NN is discerning patterns within the data at individual points. (**b**) Showcases a comparison between the NN's approximation and the actual spectrum in the through port, along with the nearest training samples. (**c**) Corresponding comparison between the NN's approximation and the actual spectrum in the drop port. Specifically, one training example represents the closest larger structure to the desired one, while the other represents the closest smaller structure.

Upon completion of the training process, the weights of the NN were stored in files for easy retrieval and utilization. To further explore the performance of the DL method in accurately estimating the transmission spectrum, we conducted a detailed investigation. The training loss graph, depicted in Fig. 3a, illustrates the performance of our NN throughout the training process. This graph reflects the network's capability to closely predict transmission spectra, resulting in a low loss value of 4 × 10^−4^. We then proceeded to evaluate the network's ability to approximate spectra that were not part of the training process. Our network's generalization capability is further assessed by comparing predicted transmission spectra with actual spectra in Fig. 3b and c. This side-by-side representation allows for a comprehensive analysis of the two spectra, revealing their similarities and differences. Notably, the forward model's predicted spectrum closely resembles the actual spectra. Remarkably, the network demonstrated the ability to accurately match spectra even beyond the training set, indicating its capability to learn and generate features that were not part of its initial training data. This finding was further supported by plotting the closest samples from the training set.

The results presented in Fig. 3b and c visually unveil the network's capacity to generalize and generate previously unknown features. This observation indicates that the NN is not solely fitting the data but rather uncovering fundamental patterns and structures within the input and output data. In summary, the assessment of the forward DNN has showcased its efficacy in precisely modeling the transmission spectrum within an AOPS. The scrupulously curated training dataset, combined with the optimized architecture of the NN, has yielded a model with the capacity to predict spectral responses across a broad parameter spectrum. The network's ability to generalize, as evidenced by accurate predictions beyond the training dataset, underscores its potential in approximating nanophotonic structures. These findings establish a robust foundation for the subsequent exploration of AOPS design based on the forward NN, as elaborated in the ensuing segment.

### AOPS design based on forward DNN

Having trained our highly accurate NN model, we've effectively eliminated the need for time-consuming FDTD calculations, substantially accelerating the process (refer to Fig. S4 for details of the computation cost of FDTD). We can now input various waveguide parameters into our trained NN, obtaining predictions within minutes. Having access to an extensive dataset of spectral responses for different structures, we can easily retrieve the desired spectral response by searching among them.

The selection of the desired switching mechanism can vary depending on the design objective of the optical switch. DL, due to its ability to provide an enormous number of spectra in a short time, empowers the designer with a high level of selectivity. This enables the designer to choose an optimal structure with a high degree of confidence for various applications. For example, we direct our attention to exploring the utmost disparity between the through port and the drop port across the spectra derived from the NN. Our specific focus centers on the third telecommunications window, wherein we elucidate the switching capabilities of the RR structure. The rationale behind selecting the third window stems from its minimal optical attenuation compared to shorter wavelengths^9^. Furthermore, the decision to employ a high contrast ratio is rooted in its advantageous implications for real-world implementations of switching devices, where it fortifies the immunity against noise and detection errors, thus engendering enhanced operational robustness^88^. Following the determination of the optimal geometric parameters via DL, FDTD electromagnetic simulations were performed to minimize errors introduced by the DL approach and obtain accurate transmission spectra of the structure.

**Figure 4:**
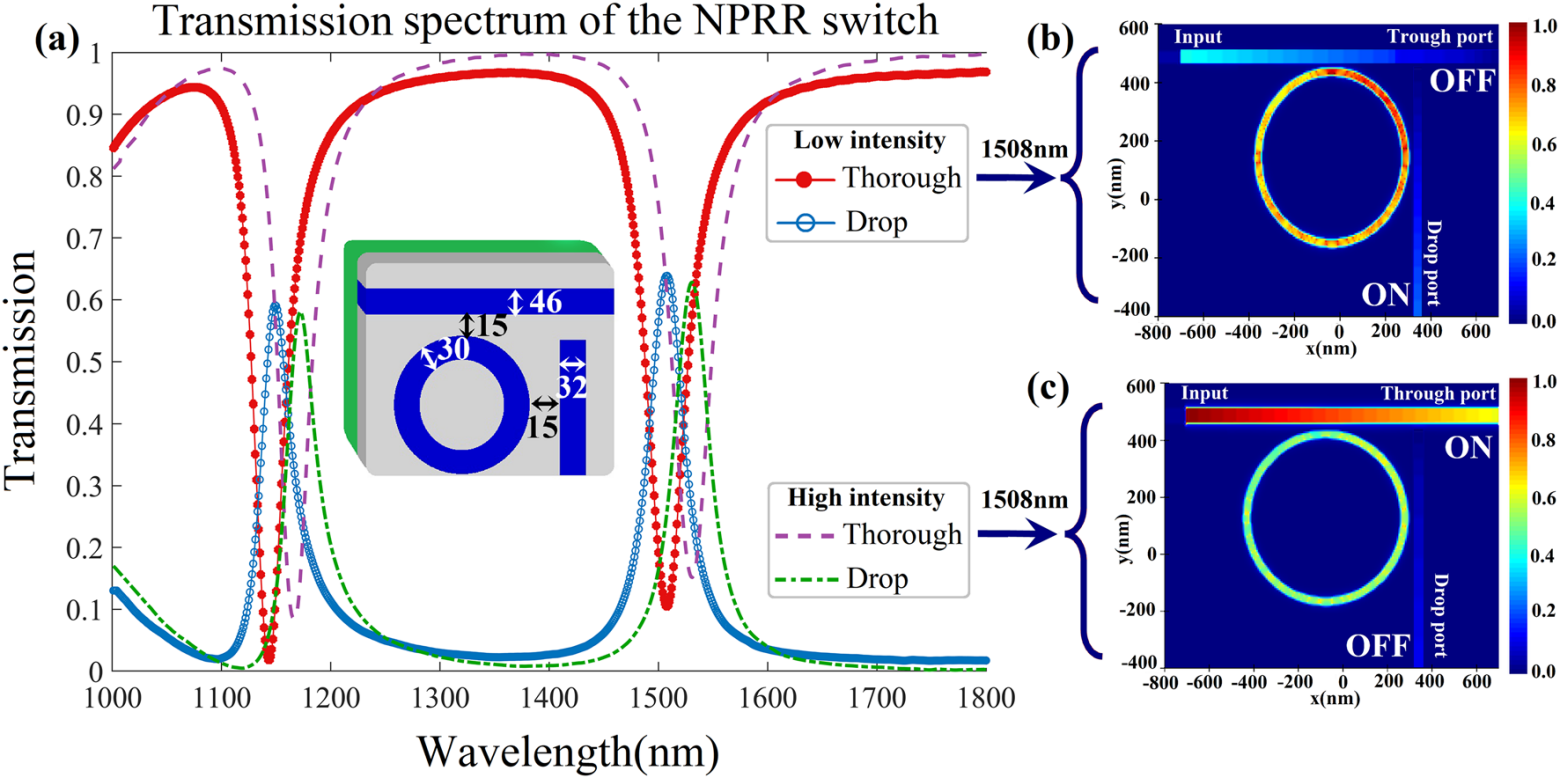
The performance of the switch device. (**a**) The transmission spectra of the circular NPRR. The resonant wavelength is observed at a wavelength of 1508 nm. As the intensity of the input light increases, the resonant wavelength undergoes a spectral redshift due to nonlinear effects. (**b**) The optical field of the AOPS under low intensity. approximately 63% of the input light passes through the drop port and 18% through the through port, indicating an "ON" state for the drop port and an "OFF" state for the through port in the AOPS (**c**) The optical field of the AOPS under high intensity. Around 58% of the input light traverses the through port and 23% through the drop port, representing an "ON" state for the through port and an "OFF" state for the drop port in the AOPS.

Figure 4a highlights optimal geometric parameters for a circular NPRR and its transmission spectrum. We analyze the device's linear and nonlinear regimes to understand its operational principles. Figure 4b and c complement the conceptual explanation, providing a direct electromagnetic simulation of the switching mechanism under both low and high optical intensities. In the linear operating regime when low-intensity light is applied at the resonant wavelength, optical power couples efficiently to the RRs, resulting in transmission to the drop port. This occurs as the resonant wavelength matches the linear resonant condition of the RRs. However, when light intensity is increased, the nonlinear Kerr effect manifests. The Kerr effect describes the phenomena wherein material refractive index changes with light intensity. Indeed, the transmission spectra exhibit a redshift due to the Kerr effect. This causes the light to become detuned from the RRs' resonance, decreasing power coupling efficiency. Consequently, instead of coupling to the RRs and transmitting to the drop port, the light passes through to the through port. The redshift induced by the Kerr nonlinearity shifts the resonance condition away from the incident wavelength, reducing light-RR coupling. This behavior demonstrates the impact of the Kerr nonlinear effect on the structure's transmission properties. By modulating light intensity, the resonance condition and power coupling to the RRs can be controlled, enabling manipulation of light routing between device ports. The Kerr effect therefore provides a means to tune light transmission in this nonlinear photonic structure.

To provide enhanced visualization of the AOPS mechanism, electromagnetic simulations were performed by applying input light pulses of varying intensity to the circular geometry at the 1508 nm resonant wavelength. Figure 4b and c plot the computed optical field patterns under low-intensity linear and high-intensity nonlinear excitation conditions, respectively. Specifically, at low input power the field profile indicates a strong resonant coupling of the signal into the rings, consistent with transmission towards the output waveguide as expected under linear regime operating principles. However, with elevated input intensity, the transmission spectrum shifts leftward due to the induced Kerr effect refractive index change. Consequently, the high-intensity field pattern in Fig. 4c reveals negligible coupling into the rings and instead shows the pulse primarily traversing the input waveguide undeflected, in agreement with the conceptual description of nonlinear switching actuation. Figure 4 displays a strong correspondence between the optical field patterns and the operation of the AOPS in both the “OFF” and “ON” states.

In addition to maximizing contrast, another approach for selecting the desired transmission spectrum can be a sharper dip in the transmission spectrum^9,11^. The selection of the sharper dip in the transmission spectrum is rooted in the inherent characteristics of the switching mechanism itself^89^. The need for a highly pronounced and rapid transition during the switching process necessitates the identification of a dip with a sharper slope. Our strategy for finding a transmission spectrum that exhibits this criterion is explained in detail in Sect. S5 of the Supplementary Information. Firstly, we demonstrate the correlation between the sharp dip in the transmission spectrum and its second derivative (see Supplementary Fig. S7 online). Subsequently, we employ this relationship in the dataset generated using the DL method, specifically targeting the wavelength of 1310 nm. The second telecommunication transmission window, centered around 1310 nm, was selected for this study due to possessing negligible chromatic dispersion within this bandwidth^90,91^.

**Figure 5:**
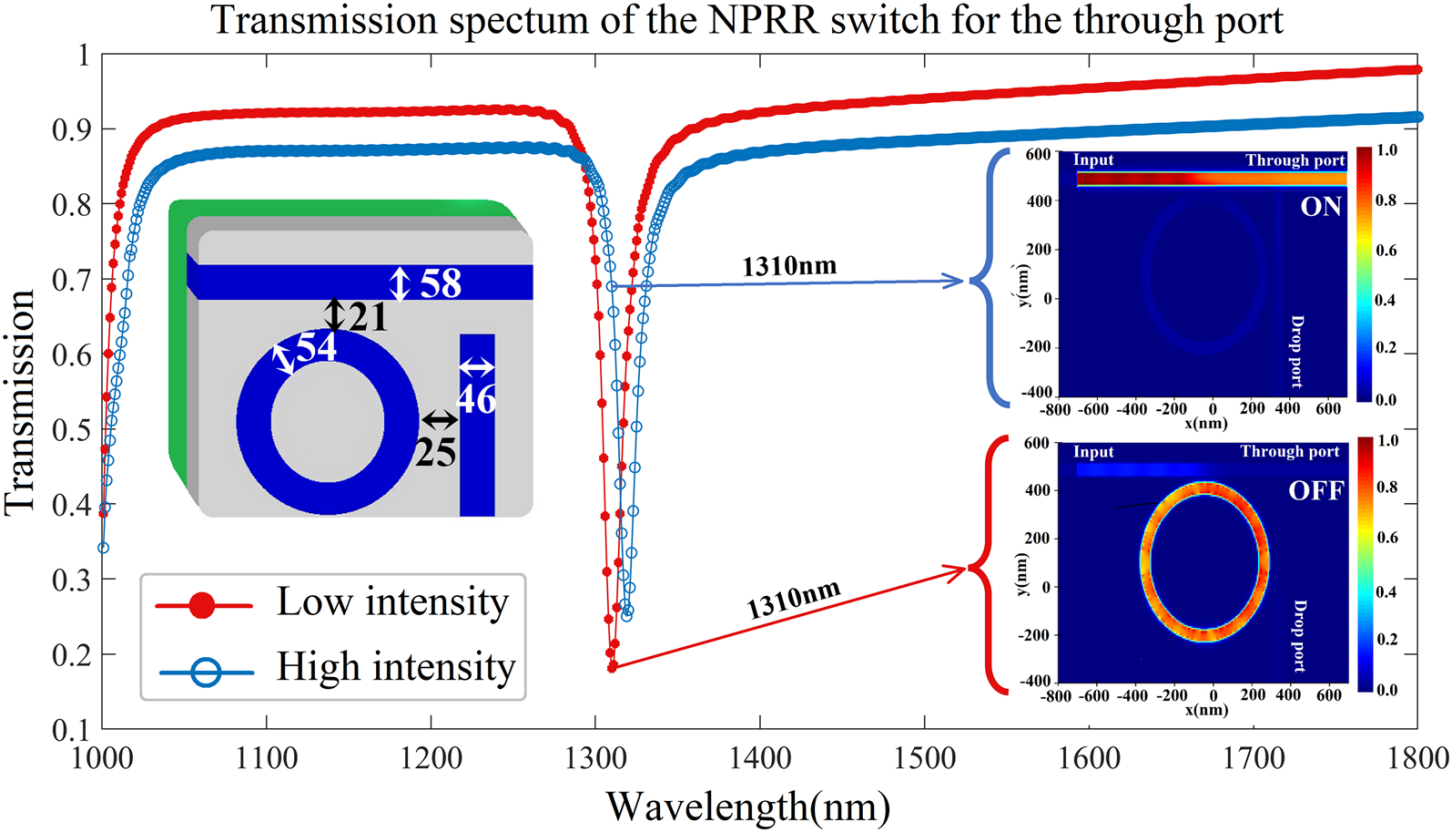
The transmission spectrum of the circular NPRR switch structures for the through port and the optical field of the AOPS in two states, under low and high-intensity conditions. The switch device's performance was assessed, by examining the transmission spectra of the optimized circular NPRR, which exhibited a resonant wavelength at 1310 nm. As the intensity of the input light increased, the resonant wavelength experienced a spectral redshift due to nonlinear effects. Furthermore, the optical field of the AOPS was illustrated. At low intensity, around 18% of the input light traversed the through port, indicating an “OFF” state for this port. Conversely, under high intensity, approximately 71% of the input light passed through the through port, signifying an “ON” state. The drop port remained in the “OFF” state in both scenarios, as the transmission through this port was negligible.

The selected transmission spectrum, determined through the proposed methodology, is shown in Fig. 5. This figure depicts the simulated transmission spectra of the proposed plasmonic switching device under conditions of low and high optical input intensities. At low intensities, when linear optical effects dominate, a clear extinction dip is visible in the transmission spectrum for through port near the resonant wavelength of the plasmonic ring. This indicates a strong coupling of the incident light into the resonant rings, in agreement with the conceptual model of the switch operating in the “ON” state. However, as the intensity is increased such that nonlinear phenomena emerge, a spectral redshift of the extinction dip is observed. This resonance shift detunes the incident light from the original resonant coupling condition, resulting in diminished power transfer to the rings and a transition to the “OFF” state as indicated by the transmission profile. These simulation results provide valuable validation of the conceptual model and illustrate how intensity-dependent modulation of the resonant properties facilitates optical switching functionality in the designed plasmonic nanostructure.

By presenting these examples, we have effectively demonstrated the significant potential of the proposed DL method in identifying the optimal structure. Furthermore, we have shown that the forward model can be used for optimization purposes. To summarize, the examination of the proposed plasmonic switching device, utilizing the forward DNN, has yielded promising insights into its operational principles and design parameters. The ability to bypass time-intensive FDTD calculations through the trained NN underscores its practical utility. The investigation of optimal geometric parameters, guided by the network's predictions, further highlights the efficiency and precision facilitated by DL in the design of AOPS. As we transition to the next part, the focus will pivot toward the practical implementation and experimental validation of the predictions made by the inverse DNN. The groundwork laid here establishes the foundation for a comprehensive comprehension and application of the forward model in real-world scenarios.

### Inverse DNN

The results of our study demonstrate the effectiveness of DL in efficiently addressing inverse design problems. In inverse design, the primary goal is to generate a desired spectrum and identify the corresponding geometry that can most accurately reproduce that spectrum. Our research underscores the proficiency of NNs in achieving this objective.

**Figure 6:**
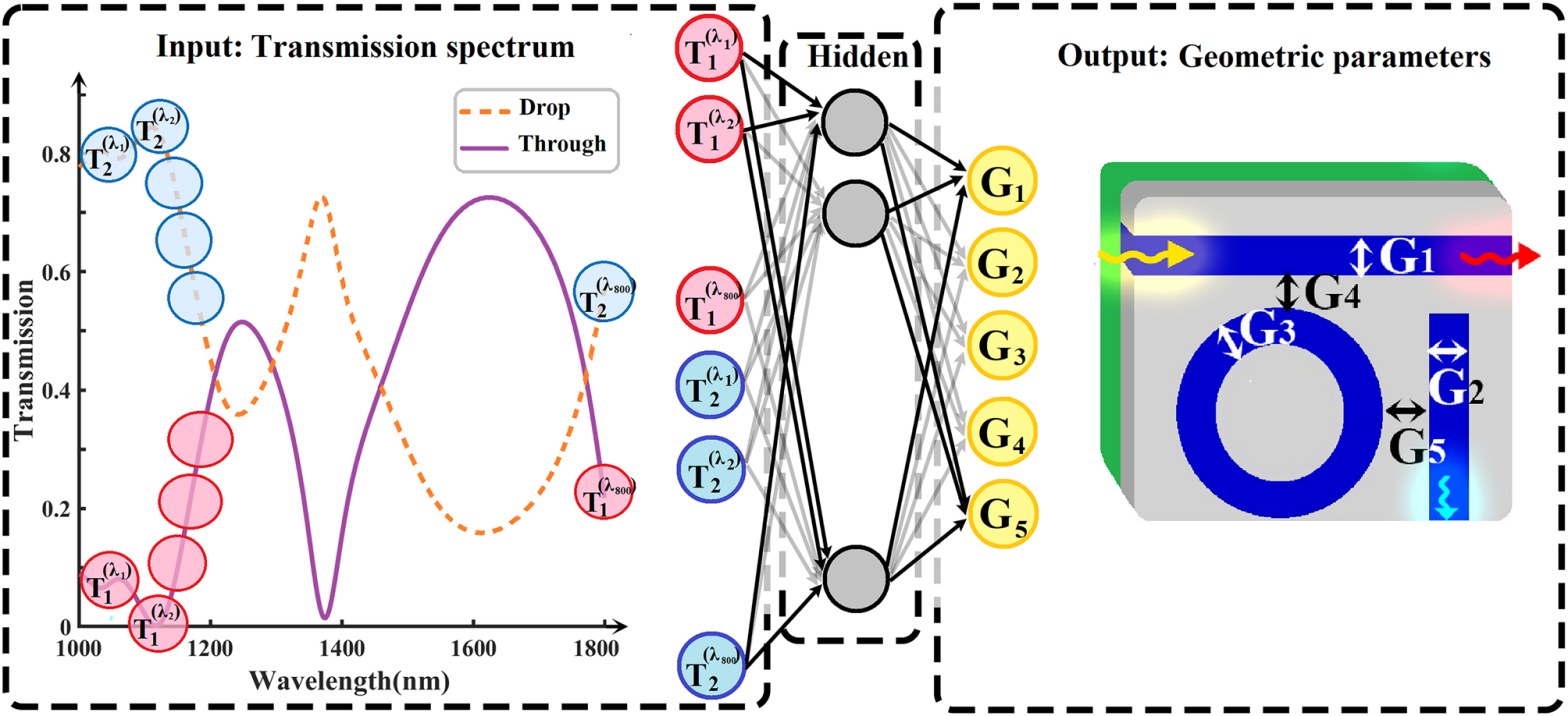
The NN architecture developed for determining the design parameters of the AOPS accepts the transmission spectra as inputs. These inputs are processed through six hidden layers, culminating in the NN producing the predicted geometric parameters, such as the waveguide width and the separation between waveguides. Such an architecture is instrumental in elucidating the intricate correlations between the transmission spectrum and the design parameters, thus offering profound understanding of the AOPS design.

The inverse model's NN is shown in Fig. 6, which takes the AOPS's Transmission spectra as an input. A considerable volume of training data facilitates the efficacious education of the DNN. The model is capable of accurately simulating and predicting a multitude of geometric parameters for AOPS configurations across a designated spectral response. Figure 6 depicts the NN's architecture, showcasing its use of spectral response data to estimate the geometric parameters of the associated AOPS. In the inverse model, we have selected an architecture akin to the forward model, which is a fully connected NN composed of hidden layers containing 6 and 60 neurons, respectively, in each layer. The selection of additional hyperparameters, which mirrors that of the forward model, is detailed in the “Method” section.

Our methodology involves selecting an arbitrary target spectrum as a reference and utilizing the trained network to predict the input parameters required to generate a spectrum resembling the target. By harnessing the predictive capabilities of the network, we can determine the exact input parameters that would generate the desired spectrum. This approach allows us to effectively utilize the network for inverse design, helping us find the required input parameters to achieve a specific spectrum. The forward and inverse operations of our system are defined by Eqs. (4) and (6), respectively. Both involve functions, **F** and **I**, which are not interchangeable but are unique to their respective processes. This distinction is clearly reflected in the distinct weight configurations of our NNs, each designed for its specific function. It is important to note that the inverse network does not perform an analytical inversion of these equations—due to the multiplicity of potential solutions in photonic design—but instead learns the statistical correlations between transmission spectra and geometric parameters. Given the complexities involved in many-to-one mappings and the need to demonstrate a meticulous distinction between forward and inverse operations, we have trained the inverse network independently. This process has been documented in the CircularRR_AOPS repository on GitHub, with separate weight files uploaded for each network.

To ensure the physical feasibility of the spectra, we derive the desired spectrum from a carefully chosen valid configuration of the AOPS. This ensures that the desired spectrum is based on a physically realistic setup, enabling us to train the network to predict input parameters that can generate similar spectra within the constraints of real-world conditions. To guarantee the robustness and reliability of the inverse model's performance, we carefully select a valid structure that maximizes the distance from the training data. This selection process ensures that the inverse model can accurately predict the desired spectrum, even for configurations that significantly deviate from those encountered during the training phase.

It is a well-known phenomenon that prediction errors by NNs tend to decrease as the proximity between test and training data increases. Conversely, widening divergence between test and training data is associated with heightened prediction errors. To evaluate a worst-case scenario, we tested the NN on “the furthest data” relative to the training data. We define “the furthest data” as the test samples exhibiting maximum distance from the training samples in feature space, based on metrics such as Euclidean distance. This methodological decision was based on the rationale that if the network displays acceptable error margins under these highly unfavorable conditions, its performance is likely to be robust across less extreme testing scenarios.

**Figure 7:**
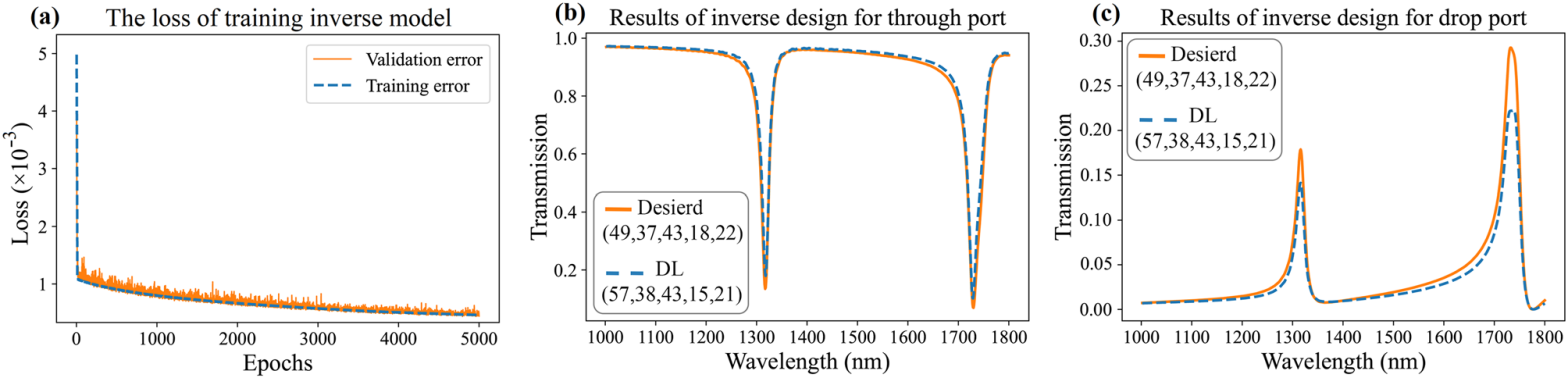
The results of inverse design for the AOPS by DL method. (**a**) The training loss is depicted, demonstrating sharp declines over time. This indicates that the NN effectively learns and discerns patterns in the data during the training process. (**b**) and (**c**) The performance of the inverse model in predicting design parameters for the transmission spectrum of the furthest data point from the training data. The legend provides the values of the geometric parameters employed in the study. The solid orange curve represents the desired spectrum, while the dashed blue curve represents the spectrum of the predicted structure generated by the DL method.

Figure 7a illustrates the loss graph during the training process, providing an overview of the inverse model's performance. This graph illustrates the network's proficiency in accurately predicting geometric parameters, yielding a minimal loss value of 1.5 × 10^−4^. While the small validation error observed in Fig. 7a is indeed promising, it alone does not suffice, particularly in light of the need to consider the many-to-one issue. Photonic structures are notably prone to the issue of non-uniqueness, often termed the many-to-one problem^92,93^. This issue emerges when disparate structural designs result in optical properties that are strikingly similar. Such non-uniqueness presents a significant challenge in the context of ML algorithms, which are conventionally engineered to refine a singular mapping from inputs to outputs, based on the premise of a one-to-one relationship between them. Within this framework, each output is linked to a definitive correct solution, and the algorithm’s aim is to progressively refine its parameters to consistently and accurately identify this solution. Nonetheless, the existence of multiple viable solutions for a single input introduces a dilemma for the algorithm, leading to ambiguity in parameter adjustment. As a result, in these instances, the achievement of convergence is not guaranteed. In response to this observation, it is pertinent to highlight that Fig. 7a demonstrates a minimal deviation between the dimensions predicted by the inverse model and the actual dimensions of the structure. This evidence suggests that the many-to-one problem, while inherent in our study, does not significantly impair the accuracy or efficacy of the inverse model. Moreover, the deliberate choice of a worst-case scenario for model evaluation, as depicted in Fig. 7b and c, have been strategically undertaken to address the many-to-one issue. This approach ensures that the model's robustness and reliability are assessed under the most challenging conditions, where the phenomenon of multiple potential solutions from a single input is most pronounced. Despite the occurrence of the many-to-one problem in this instance, the simulation results of the structural spectrum proposed by the inverse model indicate that the discrepancy between the spectra of these two structures is remarkably slight. Consequently, this mitigates the concern regarding the impact of the many-to-one problem on our model's performance.

Acknowledging that our prior explanations may not fully assuage concerns about the inverse model's efficacy in handling the many-to-one dilemma, we have embarked on a thorough evaluation. This involved testing our model against an extensive set of real-structure spectra. The model's predictions were utilized to conceptualize physical designs, which were subsequently re-simulated to yield new spectra. A comparative analysis between the original input spectra and the actual spectra of the designs suggested by the inverse model was conducted. This process enabled us to gauge the precision and dependability of our model. Detailed findings of this rigorous statistical analysis are presented in Sect. S7 of the Supplementary Information, offering an in-depth insight into the model's capability to mitigate the many-to-one issue.

In addition, Fig. 7b and c display the transmission spectra of the desired AOPS structure and the predicted transmission spectrum obtained using the DL method. These two spectra exhibit negligible differences, indicating that the DL technique successfully predicts the desired transmission spectrum with a high level of accuracy. It is worth pointing out that in solving the inverse problem, we employed the FDTD method to obtain the spectra in Fig. 7b and c, avoiding the involvement of forward model errors. The actual structure and the DL-generated structure are distinct; however, their transmission spectra exhibit high similarity. It was compelling to discover that an alternative structure could exist with a transmission spectrum approximating that of the desired structure with negligible deviation. Given that realizing the target transmission spectrum is imperative for designers of optical switches, this inverse model can readily facilitate their aims.

In this conclusive segment, we unveiled the transformative potential of the Inverse DNN in reshaping the landscape of all-optical switch design. DL can prove to be a valuable instrument in the optimization of real-world nanophotonic structures, as it adeptly addresses inverse design problems. Moreover, this method allows us to solve inverse design problems without the need for manual derivation and calculation of inverse equations. Our foray into the challenges of inverse design and the efficacy of NNs in generating desired spectra signifies a paradigm shift in nanophotonic engineering. The scrupulous processes of training and validation have bolstered the reliability of our inverse model, demonstrating its proficiency in predicting intricate geometric configurations to achieve target transmission spectra.

Our model is designed to make optimal estimations from known data to predict outcomes even when exact real-world examples are unavailable. The robust predictions of our model can withstand lack of direct empirical evidence. Additional validations of our model across a range of hypothetical scenarios, where fully realistic or complete data was unattainable, have affirmed the consistency and resilience of the model's predictions even absent empirical data. We have documented these corroborative validation findings in Sect. S6 of the Supplementary Information for your review. Figure S7 demonstrates the efficacy of the inverse model in deriving design parameters from the transmission spectrum associated with invalid data, indicating that the targeted spectrum does not align with any actual AOPS configurations. As observed in Fig. S7, the NN outputs the best approximation it can ascertain.

The inherent tangible constraints of the physical world inevitably present challenges uncaptured by mathematical or theoretical constructs. The domain of nanophotonics within the physical sciences is no exception to such discrepancies. One can articulate this discrepancy by noting certain theoretically conceived frequency responses may not correspond to any feasible physical arrangement of the device in question. Consequently, the performance of our DL model has been validated using spectra derived from a physically realistic setup. This substantiates the appropriateness of the suggested switches utilizing ANNs for integration in plasmonic circuits and underscores the practical applicability of our findings in diverse real-world scenarios.

As we delve into the strength nuances of our findings, it is essential to subject our approach to a critical lens, acknowledging inherent limitations. Notably, the performance of the DNN is intricately linked to the quality and diversity of the training dataset. The utilization of FDTD simulations for dataset generation introduces a degree of simulation-specific bias. Although experimental data often outperforms simulated data and contributes to optimal outcomes in an NN, it is crucial to recognize that the principal challenge in deploying DL-based methods lies in the collection of data. Moreover, the interpretability of the model's decisions remains a challenge, prompting avenues for further exploration into model explainability within the context of nanophotonic systems. While the model performs adequately in predicting unseen scenarios that deviate from empirical data, it should be noted that in the context of our work, if the examined spectrum diverges substantially from those considered physically plausible, the predictions made by the NN would become irrelevant for photonic engineers. Such outcomes would fail to further the practical design of AOPS and thus not address the inherent challenges within the field. As we conclude this study, we contemplate the broader implications of our findings and we anticipate that the outcomes elucidated in this article will catalyze subsequent and more impactful research endeavors by scholars in this field. The Inverse DNN not only emerges as a time-saving tool in nanophotonic design but also as a stimulant for unlocking new frontiers in optical communication.

## Conclusions

This study successfully applies DL techniques to establish a robust correlation between spectroscopic insights and the behavior of a plasmonic circular RR. Our method employs DL for predicting spectra, solving inverse design challenges, and optimizing the performance of AOPS based on the NPRR. Our NN architecture consists of six hidden layers, each comprising 60 neurons, and the training process is conducted over 5000 epochs. These specific values are carefully chosen to strike a balance between rapid convergence and accurate estimation of spectral values for the input geometrical dimensions of the AOPS. It is important to note that these values can be adjusted based on the specific problem being addressed. In real-world applications where the results may not be known in advance, it is advisable to use more conservative values for these parameters to ensure the reliable and safe performance of the DL model.

The transmission spectrum predicted by ANNs exhibits remarkable proximity to the results obtained through FDTD simulations, ensuring exceptional precision. Our DL method substantially mitigates computational expenses when contrasted with conventional FDTD solvers, enabling expeditious and cost-effective spectral estimation for RR structures. Furthermore, our approach effectively addresses the long-standing challenge of the inverse problem in designing optimal geometries for desired optical response spectra. Crucially, the performance of our DL model has been validated using spectra derived from a physically realistic setup. This substantiates the appropriateness of the suggested switches utilizing ANNs for integration in plasmonic circuits and underscores the practical applicability of our findings in diverse real-world scenarios.

The amalgamation of nanotechnology's precision and control with the computational prowess and pattern recognition capabilities of artificial intelligence holds great promise for pioneering advancements in diverse domains. This convergence is poised to catalyze a paradigm shift in scientific research, culminating in innovative applications and transformative breakthroughs across a multitude of disciplines.

### Supplementary Information

Supplementary Information.

## Data Availability

The data obtained and analyzed throughout the research can be readily accessed via the GitHub repository by utilizing the provided link: https://github.com/ehsan20e20e/CircularRR_AOPS/releases/tag/1. For supplementary data associated with the study, including the manuscript and Supplementary Information, the GitHub repository can be referenced: https://github.com/ehsan20e20e/CircularRR_AOPS. All the data are provided and made available for access.
