# Supplementary material for: A deep learning method for empirical spectral prediction and inverse design of all-optical nonlinear plasmonic ring resonator switches
Source: Sci Rep. 2024 Mar 9;14:5787. doi: 10.1038/s41598-024-56522-3 (PMC10924975; doi:10.1038/s41598-024-56522-3)
Supplement: Supplementary file 1 — Supplementary Information. [file 41598_2024_56522_MOESM1_ESM.pdf]

scientific reports

Supplementary Information for

A deep learning method for empirical spectral prediction and inverse design of all-optical nonlinear plasmonic ring resonator switches

Ehsan Adibnia,[1] Mohammad Ali Mansouri-Birjandi[1,*], Majid Ghadrdan[1], and Pouria Jafari[1]

[1]Faculty of Electrical and Computer Engineering, University of Sistan and Baluchestan (USB), P.O. Box 9816745563, Zahedan, Iran

*Corresponding author: mansouri@ece.usb.ac.ir

This PDF file includes:

Sections S1 to S7
Figures S1 to S8
References

Section S1: Theorical Segments
Electromagnetic theory

The determination of the electronic transition in metals relies on the application of the Drude-Lorentz model. This model effectively describes the behavior of free electrons in metals and their contribution to surface plasmon resonance. It is particularly suitable for materials with a higher proportion of free electrons compared to bound electrons. Drude's free-electron theory is employed to calculate the dielectric constant, which can be decomposed into its real and imaginary components[1]. The dielectric constant can be expressed as:

$$\varepsilon_{real} = 1 - \frac{\omega_p^2 \tau^2}{1 + \omega^2 \tau^2} \quad (S1)$$

$$\varepsilon_{imag} = \frac{\omega_p^2 \tau}{\omega(\omega + \omega^2 \tau^2)} \quad (S2)$$

For metals in the near-infrared frequency range, where the angular frequency (ω) greatly exceeds the inverse of the relaxation time (1/τ), the Johnson and Christy approach[1] is utilized to simplify Eq. (S1) and Eq. (S2) The simplified form of the dielectric constant is given by:

$$\varepsilon(\omega) = 1 + \frac{\omega_p^2}{\omega^2} + j\frac{\omega_p^2}{\omega^3 \tau} = \varepsilon_{real} + \varepsilon_{imag} \quad (S3)$$

In this specific scenario, the complex relative permittivity ($\varepsilon(\omega)$) is characterized by its real part (ε_{real}) and its imaginary part (ε_{imag}), while the complex refractive index (n) consists of its real part (n) and its imaginary part (κ). These quantities are related to each other as follows[2]:

$$\varepsilon_{real} = n^2 - \kappa^2 \quad (S4)$$

$$\varepsilon_{imag} = 2n\kappa \quad (S5)$$

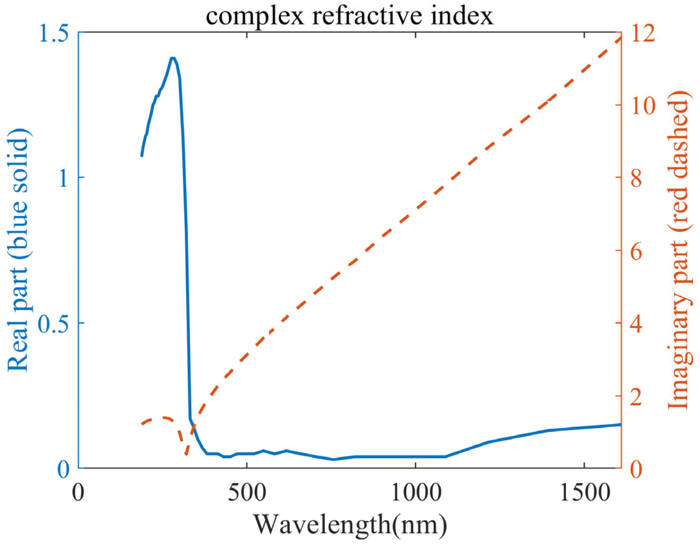

Figure S1. The data obtained from Johnson and Cristy, represent the real (blue solid curve) and imaginary (red dashed curve) components of the complex refractive index for silver. The real part of the refractive index provides insights into the phase shift experienced by light traversing through silver, while the imaginary part, also known as the extinction coefficient, characterizes the absorption and attenuation of light within the material. The comprehensive analysis of these components enables the computation of essential optical parameters, such as transmission and reflection coefficients, which further contribute to the advancement of research in the field of optics and materials science.

The real and imaginary components of the complex refractive index, obtained using the Johnson and Christy approach, are illustrated in Fig. S1. These components are subsequently utilized for the calculation of transmission and reflection coefficients.
To investigate the inherent physical plasmonic characteristics, we employ the Finite-Difference Time-Domain (FDTD) technique as a computational framework for resolving the classical Maxwell's equations. This analytical process duly considers the sinusoidal time dependence of the electric field, which is elegantly articulated as $E(r,t) = E(r)e^{-j\omega t}$. Through the derivation of the Helmholtz equation from the foundational Maxwell's equations, the ensuing mathematical expression manifests as follows:

$$\nabla^2 E + k_0^2 \varepsilon E = 0 \quad \text{(S5)}$$

Where the wavevector is denoted as k_0, and the propagating field is defined by the equation $E(x,y,z) = E(x,y)e^{j\beta z}$, where β represents the propagation constant. This propagation constant, in a complex form, is expressed as $\gamma = \alpha + j\beta$, with α characterizing the attenuation constant. When α equals zero, the complex term, $\gamma = j\beta$ delineates the sole dependency of propagation in the z-direction.

FDTD simulation

We use the FDTD numerical method to examine both linear and nonlinear behaviors of the suggested configurations. It enables us to precisely simulate the optical response of the system. In our simulations, we have chosen the mesh dimensions as $\Delta x = \Delta z = 0.5$ nm, which ensures a high level of spatial resolution. To mitigate the undesirable effects of reflections at the simulation domain boundaries, we employ a Perfectly Matched Layer (PML) as an absorptive boundary condition. The PML is highly effective in absorbing outgoing waves, thereby minimizing reflections and enhancing the accuracy of our results. The investigated architecture of the all-optical plasmonic switch (AOPS) comprises a silver film that incorporates a low refractive index Au/SiO2 layer. This specific design allows for the manipulation of SPPs and facilitates the desired switching behavior. The cladding layer surrounding the structure is composed of air, while the substrate is made of glass. These material choices are commonly employed in plasmonic devices due to their favorable optical properties.
The selection of a 50 nm thickness for both the silver film and Au/SiO2 layer was primarily driven by the optimization of the device's performance, taking into account the impact of fabrication techniques on its functioning. The inclusion of a 100 nm glass substrate served the purpose of providing mechanical support and stability to the overall structure. Through the utilization of the FDTD method, the linear and nonlinear responses of the complex AOPS configuration could be meticulously examined. This numerical approach allows for an in-depth exploration of the system's optical behavior, leading to the extraction of valuable insights and a comprehensive understanding of its unique characteristics and emergent behaviors under various conditions.

Section S2: Dataset discussion

This section provides an overview of the training dataset utilized in training the deep neural network (DNN). The training dataset plays a critical role in uncovering hidden patterns and enabling accurate predictions. By employing a comprehensive and precise training dataset, the DNN demonstrates its ability to process diverse inputs and apply appropriate weightings through the activation function in individual neurons. Figure S2a illustrates the input training dataset employed to train the developed neural network (NN). The dataset utilized in this study consists of 117,964,800 data points, calculated using the FDTD method. The dataset encompasses several parameters: G_1 represents the through waveguide width, G_2 represents the

drop waveguide width, G_3 represents the ring waveguide width, G_4 represents the through waveguide gap, and G_5 represents the drop waveguide gap. These parameters are visually represented by the respective blue, orange, green, red, and violet bars in Fig. S2a. In contrast, Fig. S2b showcases the output dataset used for training and making predictions with the developed DNN. This dataset incorporates spectral data obtained from the FDTD method, representing the transmission spectra for the through port and drop port. The through port transmission spectra are depicted by the blue bars, while the drop port transmission spectra are represented by the orange bars in Fig. S2b. This output dataset serves as the target for training and evaluating the performance of the DNN. As illustrated in Fig. S2, the data portrays a notably equitable and consistent distribution. The histogram showcases a uniform allocation of data points within distinct bins, signifying that both the input and output datasets are comprehensively dispersed, devoid of substantial bias or asymmetry. This equitable distribution implies dataset diversity and representativeness, which can be highly advantageous for training and enhancing the generalization capabilities of the neural network model.

Section S3: Analysis of the computational cost

Comparison of the computational cost related to NNs with various architectures.

In this section, we evaluate the performance of the developed NN while taking into account its computational cost. Generating extensive training datasets for DNNs requires significant computational resources, which poses a challenge in automatically generating supplementary data points, particularly for underrepresented regions not covered by existing datasets. This challenge becomes more pronounced when considering the computational burden and the researchers' involvement in data curation activities. Therefore, finding ways to augment datasets in a computationally efficient manner while preserving model generalizability is an important area for future research. Figure 3Sa provides a comparison of training execution times for different numbers of neurons and hidden layers. The computational cost is calculated based on one transmission spectra during DNN training, with the number of neurons adjusted for each hidden layer variation. The results show that more complex DNN architectures and increased numbers of neurons lead to higher computational overhead.

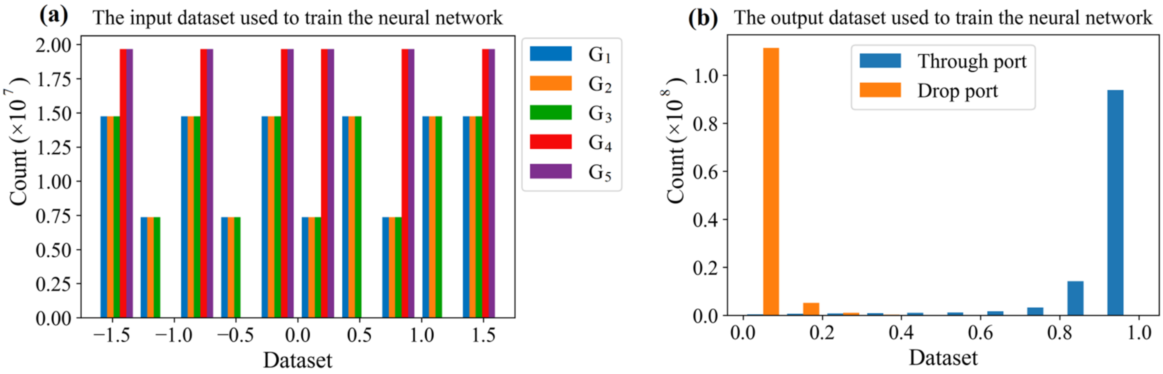

Figure S2. Histograms representing the input and output datasets for the circular ring resonator. (a) The scaled input dataset used for training the NN is depicted. This dataset comprises the G_1 = through waveguide width, G_2 = drop waveguide width, G_3 = ring waveguide width, G_4 = through waveguide gap, and G_5 = drop waveguide gap. The histogram visualizes the distribution and frequency of these input parameters. (b) The trend of the output dataset used for training the developed NN is showcased. This dataset corresponds to the transmission spectra for the through port and drop port. The histogram provides a visual representation of the distribution and characteristics of the transmission spectra for both ports.

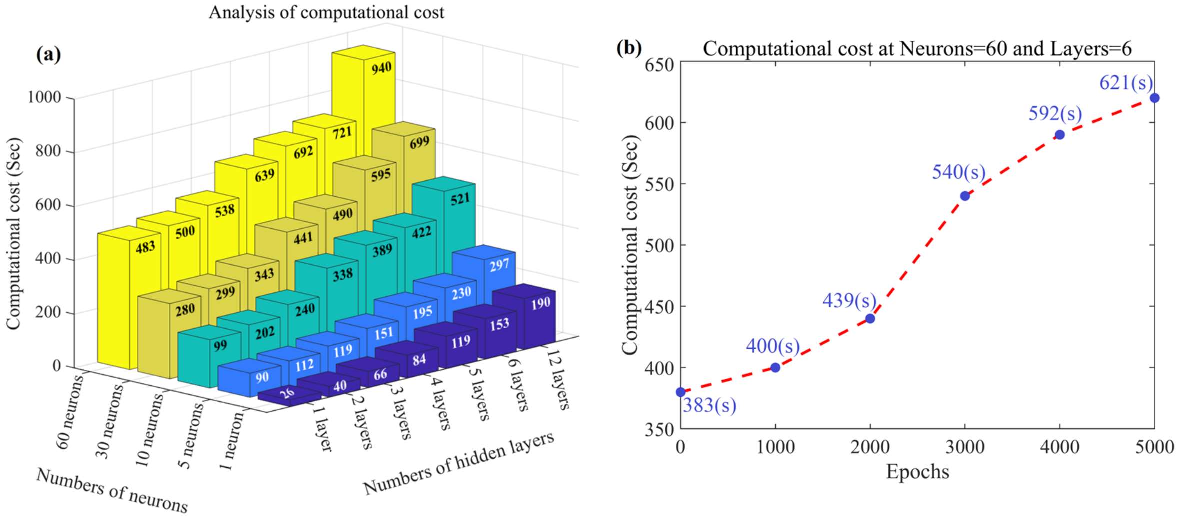

Figure S3. Comparative analysis of the computational cost of DL. (a) We present the computational cost of NNs employing diverse structures. The count of neurons is adjusted for each hidden layer variation. In such instances, a more intricate DNN and an augmentation in neuron quantities result in escalated computational overhead. Nevertheless, we observe a specific configuration, specifically six layers with sixty neurons, where incorporating additional layers and neurons fails to enhance loss and may even trigger the vanishing gradient issue, as illustrated in Fig. S5 and Fig. S6, while amplifying the computational cost. (b) The computational cost is compared for different epochs while keeping the number of neurons fixed at 60 and the number of layers fixed at 6. The results demonstrate that computational cost values tend to rise alongside increasing numbers of epochs.

However, a configuration of six layers with sixty neurons is identified where adding extra layers and neurons does not improve the loss function and may even introduce the vanishing gradient problem. Figure 3Sb demonstrates that computational cost values increase with more epochs, indicating longer processing times. Nonetheless, as delineated in Fig. S5 and S6, the forecasts produced by the DNN exhibit a closer alignment with the observed outcomes. This implies that the DNN model necessitates increased computational resources and time for achieving a convergence towards more precise predictions as the training process unfolds. Notably, the DNN's performance surpasses traditional FDTD solvers, which require thousands of seconds to compute plasmonic RR optical responses using automated meshes. While an extensive dataset created via computationally intensive electromagnetic simulations cannot be avoided, this preprocessing is a one-time investment. The trained DNN then provides rapid predictions for novel configurations, demonstrating the potential for accelerating computation tasks once training is complete. These findings highlight the trade-off between computational cost and prediction accuracy in the context of DNN architecture. It is crucial to carefully consider the available computational resources and the desired accuracy levels when selecting an appropriate configuration for a given task. Optimizing this trade-off between computational cost and prediction accuracy remains an important area for further research.

Comparison of the computational cost between DL and FDTD method.

This section aims to compare the computational cost between the DL method and the FDTD methods. In Fig. S4, we present the computational cost in seconds for the FDTD method using different meshing strategies. When employing minimum-size meshing, the computational cost amounted to 3763 seconds.

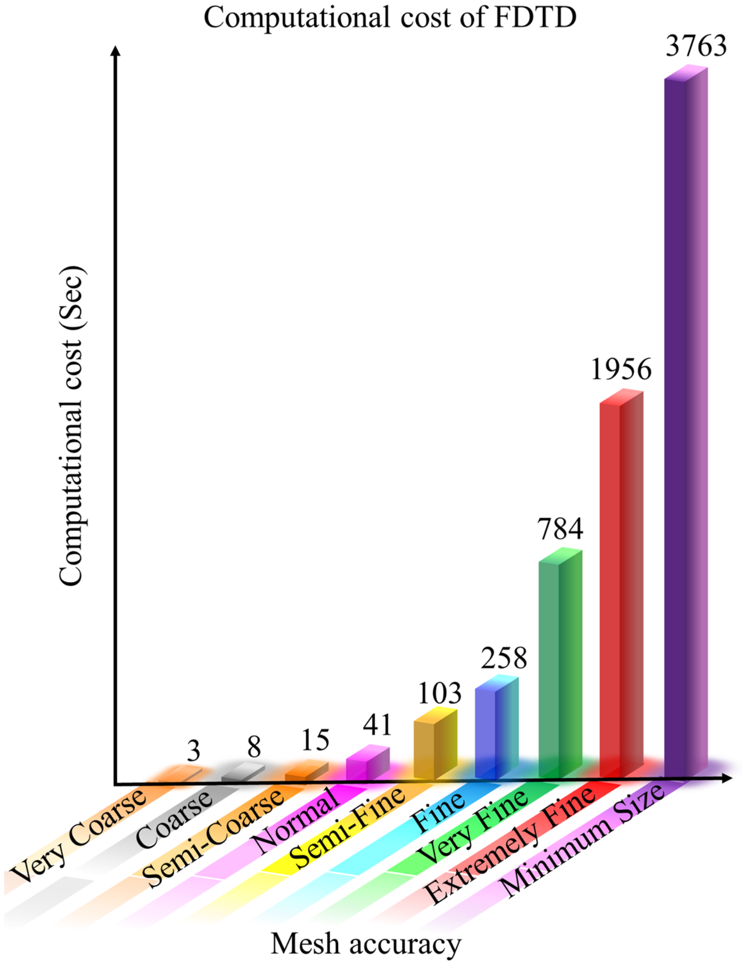

Figure S4. Comparative analysis of the computational cost of FDTD method. When employing minimum-size meshing, the computational cost for the simulation was 3763 seconds. However, when using DL techniques, the computational cost was dramatically reduced to less than one second. This significant reduction in processing time provides a considerable advantage as it results in substantial time savings for users.

This cost increased to 3 seconds, 8 seconds, 15 seconds, 41 seconds, 103 seconds, 258 seconds, 784 seconds, and 1956 seconds for very coarse, coarse, semi-coarse normal, semi-fine, fine, very fine, and extremely fine meshing, respectively. In contrast, when utilizing the DL method, the computational cost was significantly reduced to a fraction of a second. This substantial reduction in processing time is highly advantageous as it leads to significant time savings for users. In this research, we propose an alternative approach where a DNN is trained to predict spectral values, resulting in a considerable reduction in the processing time of the DL method. By amalgamating the temporal aspects of these individual components, we can deduce the total execution duration. This approach not only affords the capacity to represent more intricate scenarios in contrast to linear techniques but also offers the capability to forecast execution times for scenarios that may not have been encountered within the training dataset. Consequently, our approach not only estimates the execution time for a batch or an extended epoch but also streamlines the process of selecting the most appropriate technologies and models for a diverse spectrum of intricate and multifaceted photonic devices. This comparative analysis underscores the pronounced advantage of the DL methodology in terms of computational efficiency, especially when juxtaposed with the FDTD method employing varying meshing strategies. The reduction in processing time achieved through the DL

approach paves the way for novel opportunities in the expeditious and more resource-efficient analysis of photonic devices, ultimately fostering heightened productivity and innovation in the field.

Section S4: Optimizing hyperparameters of DNN

In this section, the focus is on optimizing the hyperparameters of a DNN to achieve the best performance in terms of mean squared logarithmic error (MSLE) between predicted and actual values. During all predictions, the learning rate was set to 0.1 to ensure optimal performance. Figure S5a presents the MSLE values for an NN with 60 neurons and 5000 epochs while varying the number of hidden layers. It becomes evident that a classical shallow network with only one hidden layer fails to perform well in the prediction task. Despite its simplicity, the shallow network is unable to capture the complex patterns and relationships present in the data. This failure highlights the limitations of shallow networks in handling intricate and nonlinear problems. On the other hand, deeper NNs, such as the one used in the study, can learn and extract more intricate features, making them more suitable for tackling challenging prediction tasks. Increasing the number of epochs leads to a reduction in MSLE values. Additionally, as the number of hidden layers increases, the MSLE values decrease and plateau at around 12 hidden layers. Based on this analysis, 6 hidden layers were chosen as the optimized hyperparameter choice, as they yielded similar MSLE values while minimizing computational load. This selection aimed to optimize the performance of the DNN and determine the most effective hyperparameters. The number of epochs was fixed at 5000, while the number of neurons was set to 60, allowing for a comprehensive evaluation of the DNN's performance. Figure S5b illustrates the impact of increasing the number of neurons on the MSLEs. Using only one neuron is insufficient for an efficient DNN. To enhance the performance of the DNN, different numbers of neurons (five, ten, thirty, and sixty) were employed. The corresponding MSLE values are represented by the green, violet, yellow, and blue curves, respectively. Based on these observations, sixty neurons were chosen for further analysis due to their lower MSLE values and faster convergence. The purpose of this selection was to optimize the DNN's performance and determine the most effective hyperparameters. We kept the number of epochs constant at 5000, while varying the number of neurons set to 60, enabling a thorough assessment of the DNN's capabilities. Fig. S5b visually demonstrates the influence of increasing the number of neurons on the MSLEs. It becomes apparent that utilizing just one neuron falls short of achieving an efficient DNN. To enhance the DNN's performance, we explored different neuron quantities, specifically five, ten, thirty, and sixty. The corresponding MSLE values are depicted by the green, violet, yellow, and blue curves, respectively. Based on these observations, sixty neurons were chosen for further analysis due to their lower MSLE values and faster convergence, underscoring their effectiveness. Initial predictions were made based on specific input geometrical dimensions, where $G_1 = G_2 = G_3 = 45$ nm and $G_4 = G_5 = 20$ nm. The corresponding transmission spectra in the through and drop ports were then predicted. Figure S6 provides a comprehensive comparison between the actual spectrum and the NN approximation for the through port and drop port analyses. In all panels of Fig. S6, the original transmission spectra obtained through the FDTD method are represented by the orange solid curve. The blue dashed curve depicts the predicted transmission spectra based on the input parameters. The left-side panels of Fig. S6 are associated with the through port analysis, while the right-side panels are associated with the drop port analysis. Figures S6a and b, S6c and d, S6e and f, S6g and h, S6i and j, S6k and l, and S6m and n illustrate the transmission spectra for different numbers of hidden layers (1, 2, 3, 4, 5, 6, and 12, respectively). These panels provide visual comparisons between the actual and predicted transmission spectra for each case.

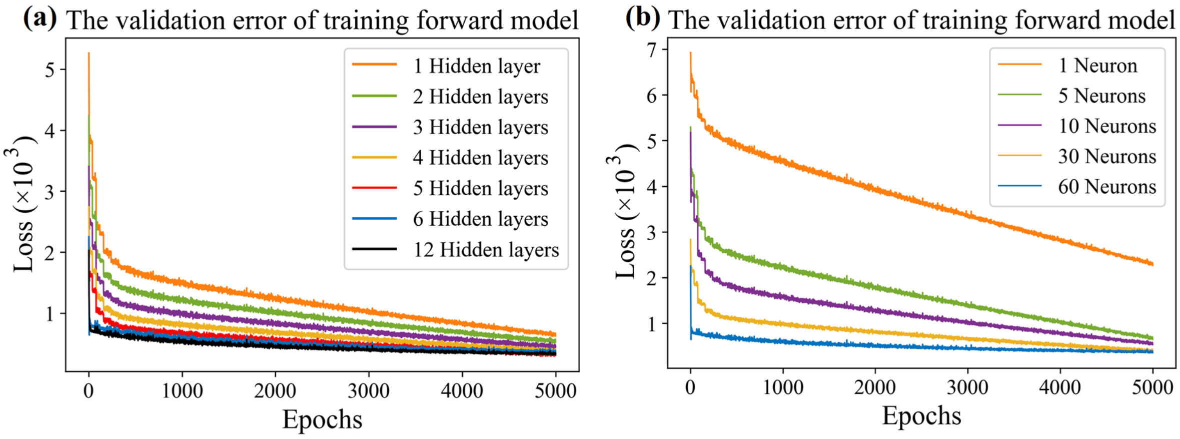

Figure S5. The loss values (Validation Set) for various configurations of hidden layers and neurons (a) the MSLE values for different numbers of hidden layers when the number of neurons is held constant at 60. Specifically, MSLE is reported for models with 1, 2, 3, 4, 5, 6, and 12 hidden layers. Shallow networks may have limitations in capturing complex patterns, while deep networks have the potential to perform better by leveraging their ability to learn hierarchical representations and extract more meaningful features from the data. (b) The MSLE values for different neurons. Specifically, 1, 5, 10, 30, and 60, when the number of neurons is set to 6. A single neuron may be inadequate for complex tasks, while increasing the number of neurons, such as utilizing sixty neurons, can enhance the DNN's performance by enabling it to capture more intricate patterns and relationships in the data.

By carefully optimizing the hyperparameters, such as the number of hidden layers and neurons, the performance of the DNN can be enhanced. This iterative process ensures that the model learns and captures the underlying patterns and relationships within the input data.
The reasons for choosing MSLE are briefly as follows:

- The MSLE was deliberately chosen as the loss function for our model due to its sensitivity to relative rather than absolute errors, which is more aligned with the bounded range of transmission values in our study.
- The MSLE is particularly adept at handling values that are clustered within a narrow interval, as it disproportionately penalizes underestimations of small true values—a critical consideration in photonic design where underestimating transmission could result in designs failing to meet efficiency thresholds.
- This loss function mitigates the undue influence of outliers, which can distort the MSE and lead to an inaccurate assessment of model performance across the majority of data points.
- The training process benefits from the more stable gradient descent optimization provided by MSLE, which tempers the effect of large error terms during backpropagation, leading to smoother convergence and enhanced model stability.
- Given the multiplicative and exponential relationships often present in photonic systems, the logarithmic nature of the MSLE aligns with the exponential dependence of transmission on design parameters, thus capturing the underlying physical dynamics more effectively.

The resulting predictions provide valuable insights into the behavior of the system under investigation. Overall, this section demonstrates the importance of hyperparameter optimization in DNNs and showcases the effectiveness of the chosen hyperparameters in accurately predicting transmission spectra.

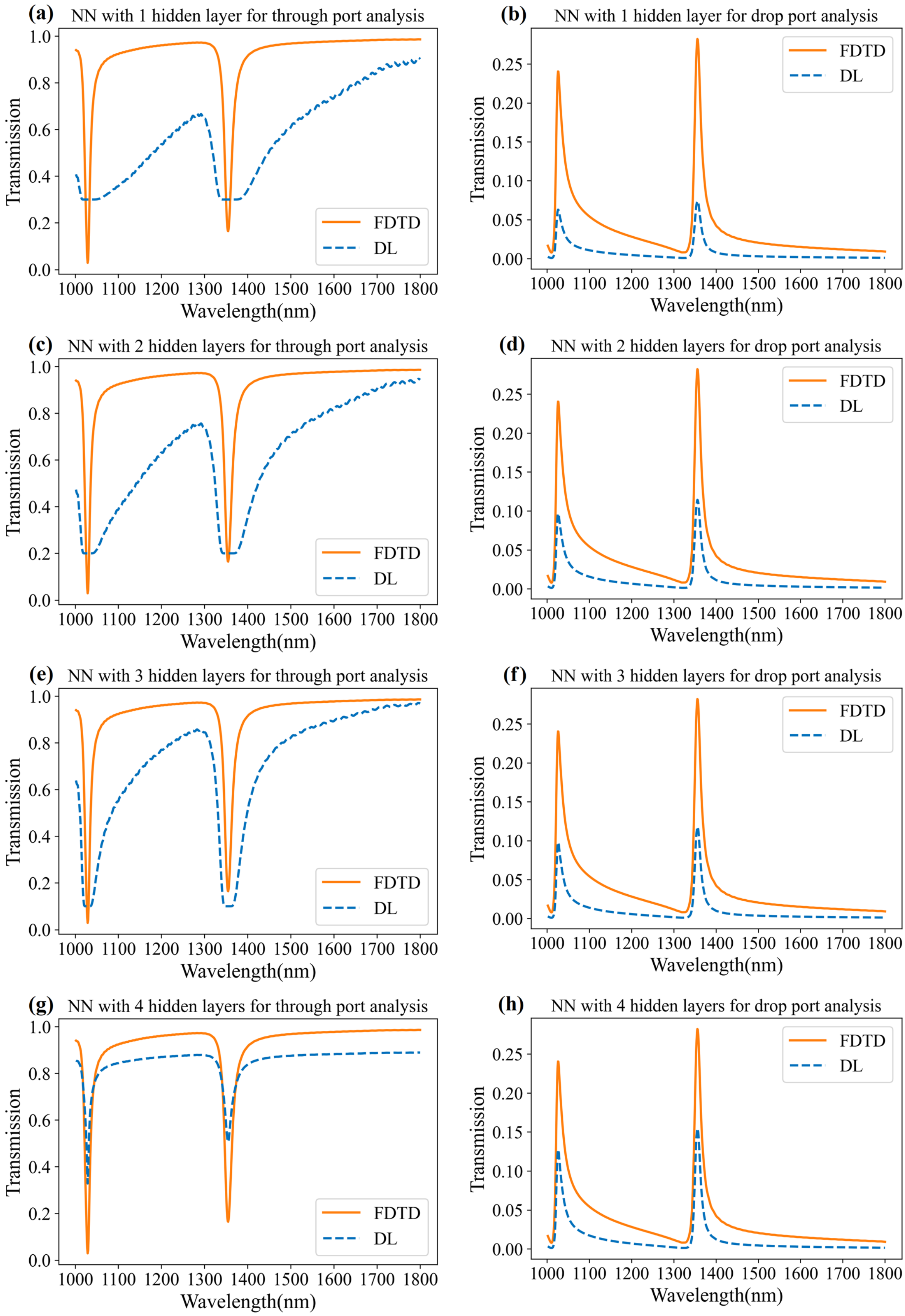

(a) NN with 1 hidden layer for through port analysis
(b) NN with 1 hidden layer for drop port analysis
(c) NN with 2 hidden layers for through port analysis
(d) NN with 2 hidden layers for drop port analysis
(e) NN with 3 hidden layers for through port analysis
(f) NN with 3 hidden layers for drop port analysis
(g) NN with 4 hidden layers for through port analysis
(h) NN with 4 hidden layers for drop port analysis
Transmission
Wavelength(nm)
FDTD
DL
1000 1100 1200 1300 1400 1500 1600 1700 1800
0.0 0.2 0.4 0.6 0.8 1.0
0.00 0.05 0.10 0.15 0.20 0.25

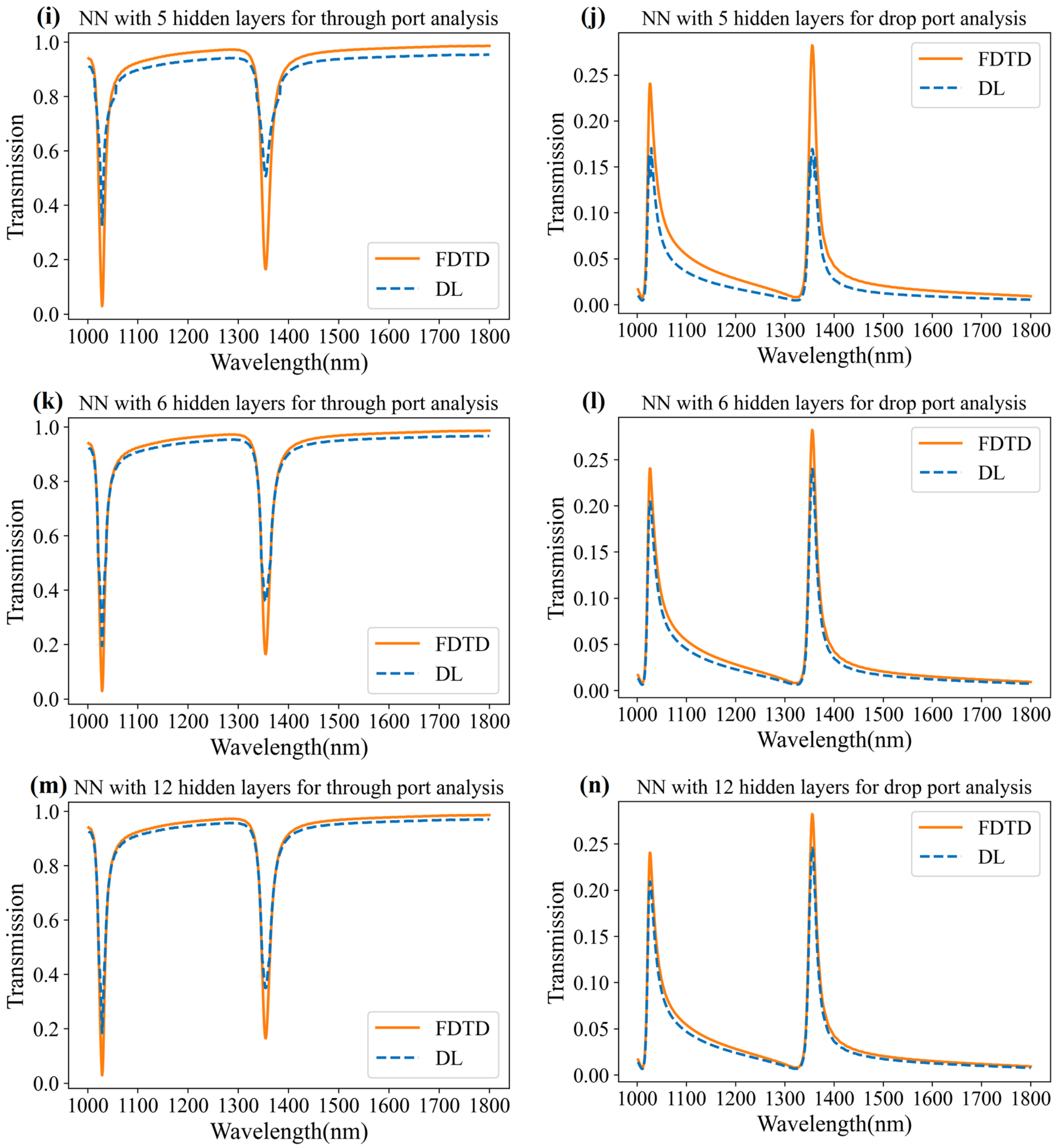

Figure S6. Comparative Assessment of Deep Learning (DL) Approximations and Original Spectra in Various Configurations: (a) Single hidden layer configuration for the through port analysis. (b) Single hidden layer configuration for the drop port analysis. (c) Two hidden layers configuration for the through port analysis. (d) Two hidden layers configuration for the drop port analysis. (e) Three hidden layers configuration for the through port analysis. (f) Three hidden layers configuration for the drop port analysis. (g) Four hidden layers configuration for the through port analysis. (h) Four hidden layers configuration for the drop port analysis. (i) Five hidden layers configuration for the through port analysis. (j) Five hidden layers configuration for the drop port analysis. (k) Six hidden layers configuration for the through port analysis. (l) Six hidden layers configuration for the drop port analysis. (m) Twelve hidden layers configuration for the through port analysis. (n) Twelve hidden layers configuration for the drop port analysis.

Section S5: The strategy for finding a sharper dip in the transmission spectrum

Within this section, we introduce and assess an approach aimed at identifying a transmission spectrum featuring a more pronounced dip. In our quest for a more desirable spectral response, our focus is directed towards the second-order derivative of the transmission spectrum, mathematically represented as $\mathrm{d}^2x/\mathrm{d}\lambda^2$. As portrayed in Fig. S7, a heightened peak in the second derivative plot (Fig. S7a) corresponds to a sharper dip in the transmission spectrum (Fig. S7b). This clear correlation between the second derivative plot and the transmission spectrum offers a deeper insight into the spectral characteristics, thereby facilitating the design and optimization of nanophotonic structures.

For this example, we have plotted the transmission spectra of five consecutive structures. To mitigate potential errors introduced by the DL method, we acquired the transmission spectra through the FDTD method for this figure.

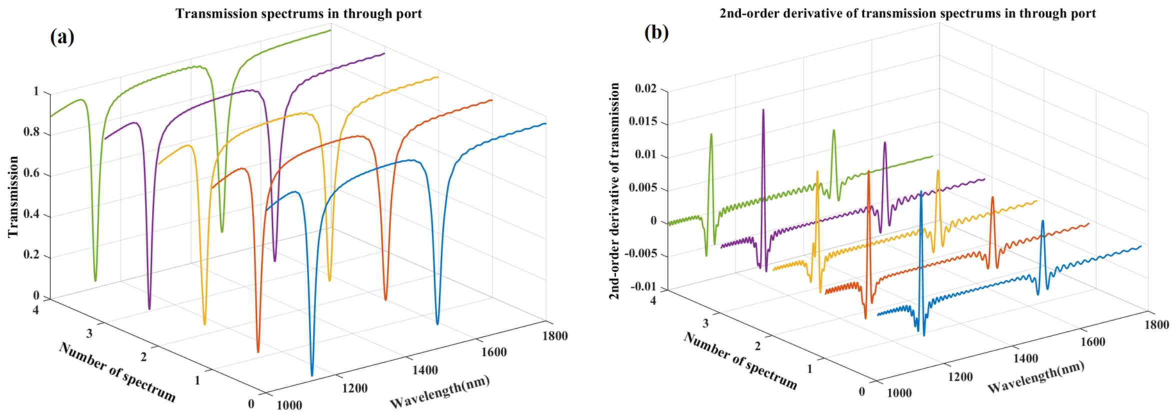

Figure S7. Illustrating the Correlation Between Sharp Dips in the Transmission Spectrum and Its Second Derivative: (a) Depicts the transmission spectrum. (b) Represents its second-order derivative. As evident, a more prominent peak in the second derivative plot signifies a sharper dip in the transmission spectrum

Section S6: the model's predictions' consistency and resilience in the absence of empirical data

Within this section of our study, the inverse model is subjected to evaluation using spectral data not derived from genuine physical AOPS constructs. To this end, an artificial spectrum from an indeterminate structure has been input into the NN to represent the target spectrum, as depicted in Fig. S8. The efficacy of the neural network was remarkable; it deduced a structure from the synthetic spectrum which, upon subsequent verification via FDTD simulations, demonstrated significant congruence with the target spectrum. In essence, the NN adopts the nearest feasible approximation as its output.

Section S7: Addressing the many-to-one problem

In this segment of our study, we curated a dataset comprising 5,000 authentic AOPS structures, subsequently determining their spectra via an FDTD solver. These spectra were then inputted into the inverse model, which utilized DL-based methodologies to predict corresponding structures. In the concluding phase, we employed the FDTD solver again to simulate the spectra of these predicted structures. This allowed us to compute the mean error between the simulated and the predicted spectra across the 5,000 structures. The resultant average error was found to be 0.04, signifying that the many-to-one issue does not markedly detract from the effectiveness

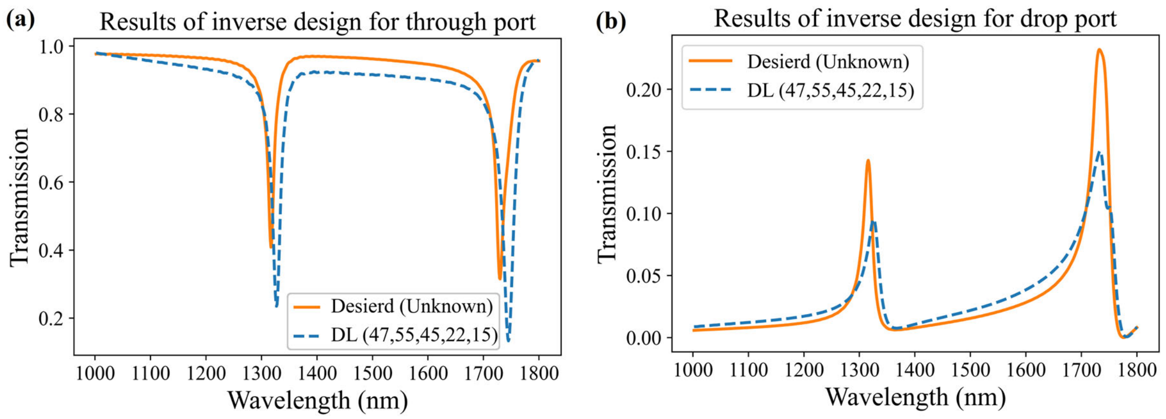

Figure S8. The inverse model's performance to estimate design parameters from the transmission spectrum of invalid data is assessed, where the intended spectrum does not correspond to any tangible AOPS structures. The legend delineates the geometric parameters utilized within the research. The solid orange curve illustrates the hypothetical desired spectrum, whereas the dashed blue curve depicts the spectrum of the structure projected by the DL technique.

of our approach. Given that the spectra predicted by the inverse model closely match the spectra envisioned by the designer, the design process of AOPS encounters no significant obstacles. The strong alignment between the predicted and desired spectra suggests that the inverse model effectively supports the AOPS design process without introducing any complications.

The details for calculating the average error of 0.04 is outlined as follows:

The discrepancy between predicted and actual spectra was evaluated using the Root Mean Square Error (RMSE), selected for its capability to sensitively detect significant discrepancies, thereby offering a scale-appropriate metric of predictive accuracy.

For a dataset encompassing 5,000 AOPS structures, the RMSE was determined by contrasting the spectrum forecasted by our inverse model with that obtained from FDTD simulations of the envisaged structures. The RMSE was computed as follows:

$$RMSE = \sqrt{\frac{1}{800}\sum_{i=1}^{800}(P_i - A_i)^2} \tag{S6}$$

Where, P_i represents the predicted spectral value, and A_i the actual (simulated) spectral value, with each spectrum comprising 800 data points.

The mean error of 0.04 was derived by averaging the RMSE across all 5,000 structures, thus reflecting the overall predictive accuracy of our inverse model for the dataset.

References

1 Johnson, P. B. and Christy, R.-W. Optical constants of the noble metals. *Physical review B* **6**, 4370 (1972).

2 Wooten, F. *Optical properties of solids*. (Citeseer, 1972).
